# Supplementary material for: ‘Overnight, things changed. Suddenly, we were in it’: a qualitative study exploring how surgical teams mitigated risks of COVID-19
Source: BMJ Open. 2021 Jun 16;11(6):e046662. doi: 10.1136/bmjopen-2020-046662 (PMC8210660; doi:10.1136/bmjopen-2020-046662)
Supplement: Supplementary data [file bmjopen-2020-046662supp002.pdf]

Researcher profiles

All individuals who conducted the interviews (DE, CO, LR, MJ, JLD, SP, KNLA) have extensive experience of the application of qualitative research methods to improve the design and conduct of health services research, and each have PhDs in health-related fields using qualitative methods. All researchers are based at the University of Bristol, and are members of the QuinteT research group (which uses qualitative research methods to optimise recruitment and informed consent to randomised controlled trials, many of which are surgical) and of the Centre for Surgical Research (which aims to improve the surgical evidence base, and subsequently patient care, through high quality multidisciplinary research). JLD is a Professor of Social Medicine. DE is a Research Fellow in Qualitative Methodology Research. LR is a Senior Lecturer in Qualitative Health Sciences. MJ is a Senior Lecturer in Qualitative Health Science and Senior Research Fellow. CO is a Senior Research Associate in Health Services Research. KNLA is a Senior Lecturer in Health Services Research. SP is a Research Fellow in Qualitative Methodology Research. All researchers involved in the analysis (DE, CO, LR, MJ, JLD) have experience of conducting grounded theory methodology in multiple projects and have published their findings in peer reviewed journals (see below for examples).

Selected publications from the research team, using grounded theory approaches:

Donovan, J. (1995), The process of analysis during a grounded theory study of men during their partners' pregnancies. Journal of Advanced Nursing, 21: 708-715. https://doi.org/10.1046/j.1365-2648.1995.21040708.x

Barclay L, **Donovan J**, Genovese A. Men's experiences during their partner's first pregnancy: a grounded theory analysis. Australian Journal of Advanced Nursing. 1996 Autumn;13(3):12-24. PMID: 8717683.

Featherston K, **Donovan JL**. Random allocation or allocation at random? Patients' perspectives of participation in a randomised controlled trial. BMJ. 1998;317(7167):1177-80.

Donovan JL, Paramasivan S, de Salis I, Toerien M. Clear obstacles and hidden challenges: understanding recruiter perspectives in six pragmatic randomised controlled trials. Trials. 2014;15(1):5.

Donovan JL, Salis I, Toerien M, Paramasivan S, Hamdy FC, Blazeby JM. The intellectual challenges and emotional consequences of equipoise contributed to the fragility of recruitment in six randomised controlled trials. J Clin Epidemiol. 2014;67.

Elliott D, Hamdy FC, Leslie TA, Rosario D, Dudderidge T, Hindley R, **Donovan JL**. Overcoming difficulties with equipoise to enable recruitment to a randomised controlled trial of partial ablation vs radical prostatectomy for unilateral localised prostate cancer. BJU Int. 2018;122(6):970-7.

Husbands S, **Elliott D**, Davis T, Blazeby JM, Harrison EF, Montgomery AA, Sprange K, Dule L, Karantana A, Hollingworth W, Mills N. (2020). Pilot and Feasibility Studies, 6, 173: https://doi.org/10.1186/s40814-020-00710-1.

Jepson M, **Elliott D**, Conefrey C, Wade W, **Rooshenas L**, Wilson C, Beard D, Blazeby JM, Birtle A, Halliday A, Stein R, **Donovan JL**. (2018). Patients find it difficult to comprehend gambling-related metaphors and computer-agency descriptions of randomisation during recruitment to randomised controlled trials. Journal Clinical Epidemiology. DOI:10.1016/j.jclinepi.2018.02.018

Townsend D (**Elliott**), Reeves B, Taylor J, Chakravarthy U, O'Reilly D, Hogg R, et al. Health professionals' and service users' perspectives of shared care for monitoring wet age-related macular degeneration: a qualitative study alongside the ECHoES trial. BMJ Open. 2015;5(4).

Rooshenas L, Elliott D, Wade J, **Jepson M**, Paramasivan S, Strong S, et al. Conveying Equipoise during Recruitment for Clinical Trials: Qualitative Synthesis of Clinicians' Practices across Six Randomised Controlled Trials. PLoS Med. 2016;13(10):e1002147.

Rooshenas L, et al. Bluebelle study (phase A): a mixed-methods feasibility study to inform an RCT of surgical wound dressing strategies. BMJ open. 2016;6(9):e012635.

Zahra J, Paramasivan S, Blazeby J, Cousins S, Avery K, Blencowe N, Mathews J, **Elliott D**. Discussing surgical innovation with patients: A qualitative study of surgeons' and governance representatives' views. BMJ Open. 2020;10:e035251. doi:10.1136/bmjopen-2019-035251
