# Supplementary material for: ‘Overnight, things changed. Suddenly, we were in it’: a qualitative study exploring how surgical teams mitigated risks of COVID-19
Source: BMJ Open. 2021 Jun 16;11(6):e046662. doi: 10.1136/bmjopen-2020-046662 (PMC8210660; doi:10.1136/bmjopen-2020-046662)
Supplement: Supplementary data [file bmjopen-2020-046662supp003.pdf]

Understanding risks, mitigation and innovations for Surgery in a COVID19 world: Topic guide

Introduction

- Thank you for taking the time to talk. Know there is so much going on at the moment and will try not to take too long.
- You can stop the interview at anytime.
- Purpose of interview:
  - We know that COVID19 presents new risks in theatre and to operating.
  - Across the world all sorts of ideas are emerging to mitigate the risk and experience is accumulating.
  - The purpose of our interview study is to listen to your **views and to understand your experiences and any solutions you have found to these challenges**.
  - We find that personal views of complex situations may yield information not accessed by standard methods. We're hoping to identify useful ideas and we intend to share good practice from this study rapidly in real time (anonymised data) to help others.
- Am quali researcher at UoB (not HP – apologies if some questions seem silly).
- Everything confidential, quotes anonymous.

Check recording

- Will send/have sent written consent form but can I just confirm you're happy to take part in the interview and for me to record it?

Impact of COVID19

How are you? How things have been in hospital since the pandemic?

- Can you talk to me about how Covid19 has affected surgery? *(Consider both elective and emergency procedures).*
- Which procedures are considered 'essential' during this time? *How judged? Who/how decisions made?*
- In your opinion what are the key risks in surgery due to Covid19? How identified these risks? *(training, guidance, experience)*
- Which procedures within your specialty are at a higher risk during this time? Why?

Can you describe a case where COVID19 affected what happens in the operating theatre?

- In Covid confirmed patients, those suspected, or all?
- Which staff at risk?

PPE

I'm still getting to grips with what constitutes as PPE. In your own words, can you talk me through how PPE has been used during Covid19? (ie – masks, eyewear, full hazmat suits)

- Thoughts on these in terms of:
  - *Practicalities?*
  - *Comfort?*
  - *Communication?*
  - *Whether training provided/needed?*
  - *Availability?*
  - *Does the equipment provide sufficient mitigation to risks of spread? If not, why not?*
  - *What would you class as minimal/optimal PPE?*
  - *Are there different examples for different procedures?*
  - *Necessary for confirmed Covid19 patients, suspected, or all patients, at this time?*

Role of team

How has COVID19 affected the theatre team?

- Are staff self-isolating if COVID19 suspected? Are staff tested?
- The RCS Guidance refers to 'minimal theatre staff'. Has Covid19 affected who is present? *How do you define 'minimal? Who do you think SHOULD be there (essential/ desirable)*
- Has COVID affected theatre time? *Pre/post-op prep?*

Considering other factors

What else is important? Is there anything else do we need to know or info we should we share with others? Consider:

- Surgical approaches that may be safer/riskier? *(e.g. laparoscopic, endoscopic, open – and implications of changing techniques)*
- Pre-surgical prep?
- Handwashing?

- Location of surgery *(e.g. anteroom)*
- Affected how anaesthesia used?

Given that so much has changed because of Covid19, how will surgery be in the future?

- How to re-start surgery that has stopped?

Ending the interview

- Summarise key info – what are key lessons learned regarding safety in surgery? Anything else important?
- Thank for time
- Request additional people to interview
