# Supplementary material for: ‘Overnight, things changed. Suddenly, we were in it’: a qualitative study exploring how surgical teams mitigated risks of COVID-19
Source: BMJ Open. 2021 Jun 16;11(6):e046662. doi: 10.1136/bmjopen-2020-046662 (PMC8210660; doi:10.1136/bmjopen-2020-046662)
Supplement: Author's manuscript [file bmjopen-2020-046662.draft_revisions.pdf]

BMJ Open is committed to open peer review. As part of this commitment we make the peer review history of every article we publish publicly available.

When an article is published we post the peer reviewers' comments and the authors' responses online. We also post the versions of the paper that were used during peer review. These are the versions that the peer review comments apply to.

The versions of the paper that follow are the versions that were submitted during the peer review process. They are not the versions of record or the final published versions. They should not be cited or distributed as the published version of this manuscript.

BMJ Open is an open access journal and the full, final, typeset and author-corrected version of record of the manuscript is available on our site with no access controls, subscription charges or pay-per-view fees (http://bmjopen.bmj.com).

If you have any questions on BMJ Open's open peer review process please email info.bmjopen@bmj.com

BMJ Open

"Overnight, things changed. Suddenly, we were in it": A qualitative study exploring how surgical teams mitigated risks of COVID-19

Journal:	*BMJ Open*
Manuscript ID	bmjopen-2020-046662
Article Type:	Original research
Date Submitted by the Author:	09-Nov-2020
Complete List of Authors:	Elliott, Daisy; University of Bristol, Population Health Sciences<br>Ochieng, Cynthia; University of Bristol, Population Health Sciences<br>Jepson, Marcus; Population Health Sciences<br>Blencowe, Natalie; University of Bristol, Population Health Sciences<br>Avery, Kerry; University of Bristol, Population Health Sciences<br>Paramasivan, Sangeetha ; University of Bristol, Population Health Sciences<br>Cousins, Sian; University of Bristol, Population Health Sciences<br>Skilton, Anni; University of Bristol<br>Hutchinson, Peter; University of Cambridge, Academic Neurosurgery<br>Jayne, David; Leeds Teaching Hospitals NHS Trust<br>Birchall, Martin; University College London Ear Institute<br>Blazeby, Jane; University of Bristol, Population Health Sciences<br>Donovan, Jenny; University of Bristol, Population Health Sciences<br>Rooshenas, Leila; University of Bristol, Population Health Sciences
Keywords:	QUALITATIVE RESEARCH, SURGERY, COVID-19

SCHOLARONE™
Manuscripts

BMJ

I, the Submitting Author has the right to grant and does grant on behalf of all authors of the Work (as defined in the below author licence), an exclusive licence and/or a non-exclusive licence for contributions from authors who are: i) UK Crown employees; ii) where BMJ has agreed a CC-BY licence shall apply, and/or iii) in accordance with the terms applicable for US Federal Government officers or employees acting as part of their official duties; on a worldwide, perpetual, irrevocable, royalty-free basis to BMJ Publishing Group Ltd ("BMJ") its licensees and where the relevant Journal is co-owned by BMJ to the co-owners of the Journal, to publish the Work in this journal and any other BMJ products and to exploit all rights, as set out in our licence.

The Submitting Author accepts and understands that any supply made under these terms is made by BMJ to the Submitting Author unless you are acting as an employee on behalf of your employer or a postgraduate student of an affiliated institution which is paying any applicable article publishing charge ("APC") for Open Access articles. Where the Submitting Author wishes to make the Work available on an Open Access basis (and intends to pay the relevant APC), the terms of reuse of such Open Access shall be governed by a Creative Commons licence – details of these licences and which Creative Commons licence will apply to this Work are set out in our licence referred to above.

Other than as permitted in any relevant BMJ Author's Self Archiving Policies, I confirm this Work has not been accepted for publication elsewhere, is not being considered for publication elsewhere and does not duplicate material already published. I confirm all authors consent to publication of this Work and authorise the granting of this licence.

For peer review only

"Overnight, things changed. Suddenly, we were in it": A qualitative study exploring how surgical teams mitigated risks of COVID-19

Authors:

Daisy Elliott PhD[1], Cynthia Ochieng PhD[1], Marcus Jepson PhD[2], Natalie S Blencowe PhD[1,3], Kerry NL Avery PhD[1], Sangeetha Paramasivan PhD[2], Sian Cousins PhD[1], Anni Skilton MSc[1], Peter Hutchinson PhD FRCS[4], David Jayne MD[5], Martin Birchall MD[6], Jane M Blazeby PhD FRCS [1,3], Jenny L Donovan PhD[2], Leila Rooshenas PhD[2]

Affiliations:

[1] National Institute for Health Research Bristol Biomedical Research Centre, Surgical Innovation Theme, Centre for Surgical Research, University of Bristol, 39 Whatley Road, Clifton, Bristol, UK, BS8 2PS

[2] Population Health Sciences, Bristol Medical School, University of Bristol, 39 Whatley Road, Clifton, Bristol, UK, BS8 2PS

[3] University Hospitals Bristol NHS Foundation Trust, Bristol, UK, BS2 8HW

[4] Division of Neurosurgery, Department of Clinical Neuroscience, University of Cambridge, Cambridge CB2 2QQ

[5] Leeds Institute of Medical Research, Level 7 Clinical Sciences Building, St. James's University Hospital, Leeds, LS9 7TF

[6]Ear Institute, Faculty of Brain Sciences, UCL, London

Correspondence to: Daisy Elliott [daisy.elliott@bristol.ac.uk]

Running title: Mitigating COVID-19 risks in surgery

Keywords: Surgery; COVID-19, Risks; Qualitative

Submission type: Original article

Funding statement: This study was funded by the Royal College of Surgeons (RCS Covid Research Group) and Rosetrees Trust, with support from the NIHR Biomedical Research Centre at University Hospitals Bristol and Weston NHS Foundation Trust and the University of Bristol and NIHR Advanced Surgical Technologies Incubator. The views expressed in this publication are those of the author(s)

For peer review only - http://bmjopen.bmj.com/site/about/guidelines.xhtml

and not necessarily those of the NHS, the National Institute for Health Research or the Department of Health and Social Care. JLD and JMB are NIHR Senior Investigators. NB is an MRC Clinician Scientist.

Word count: 2978 (excluding title page, abstract, quotes and references)

For peer review only

For peer review only - http://bmjopen.bmj.com/site/about/guidelines.xhtml

Abstract:

Objectives: COVID-19 presents a risk of infection and transmission for operating theatre teams. Guidelines to protect patients and staff emerged and changed rapidly based on expert opinion and limited evidence. This paper presents the experiences and innovations developed by international surgical teams during the early stages of the pandemic to attempt to mitigate risk.

Design: In-depth, semi-structured interviews were audio-recorded, transcribed and analysed thematically using methods of constant comparison.

Participants: 43 participants, including surgeons from a range of specialities (primarily general surgery, otolaryngology, neurosurgery, cardiothoracic and ophthalmology), anaesthetists and those in nursing roles.

Setting: The UK, Italy, Spain, USA, China and New Zealand between March and May 2020.

Results: Surgical teams sought to mitigate COVID-19 risks by modifying their current practice with an abundance of strategies and innovations. Communication and teamwork played an integral role in how teams adapted, although participants reflected on the challenges of having to improvise in real time. Uncertainties remained about optimal surgical practice and there were significant tensions where teams were forced to balance what was best for patients whilst contemplating their own safety.

Conclusions: The context of surgery has changed dramatically, and surgical teams have developed a plethora of innovations. There is an urgent need for high-quality evidence to inform surgical practice that optimises the safety of both patients and healthcare professionals as the COVID-19 pandemic unfolds.

Word count: 243

For peer review only

Summary box – strengths and limitations

- Undertaking qualitative interviews provided important and rich insights into surgical teams' experiences of current practices in the United Kingdom (UK) and internationally.
- Purposeful sampling ensured that a range of participants were recruited in relation to role, specialty, and geographic location.
- Participants were predominately identified via snowball sampling, making it possible that the findings may not be representative of all healthcare professionals involved in the management and treatment of patients undergoing surgical procedures.
- Interviews were conducted during the first wave of the pandemic, so further research is warranted to continue to explore how teams continue to adapt.

For peer review only

Introduction:

The World Health Organisation declared the COVID-19 outbreak a pandemic on 11 March 2020(1). Many countries have since struggled with escalating case numbers and strained healthcare systems(2). Implementation of infection prevention and control is crucial to deliver healthcare, especially for the personal protection of healthcare workers(3, 4). Operating theatres are particularly vulnerable areas due to high-risk transmission activities such as airway management transmission, aerosol-generating procedures (AGPs) and the involvement of multiple staff(5). The additional strain presented by a high prevalence of disease, limited resources and staff under pressure greatly increase the risks of transmission and the burden on healthcare systems(6). Guidelines were rapidly published (7) (8), including recommendations from The Royal Colleges (such as the Royal College of Surgeons of England, Royal College of Anaesthetists and The Intensive Care Society), Public Health England and the Department of Health. They have been produced based on expert opinion, surveys, consensus work and rapid reviews, and continue to evolve as evidence unfolds (9).

The pandemic inevitably provoked creative solutions to mitigate the risk of infection transmission(10). Innovation can be associated with modifications to decisions around whether and how to operate, surgical techniques, tools and technology, surgical team composition and operating conditions (11). Although a handful of studies have suggested practical strategies to mitigate COVID-19 risks (2, 9, 12-14), qualitative inquiry is a valuable tool for capturing dynamic and complex responses to a pandemic (15). These methods allow us to understand the ways people make sense of what is happening around them (16). The current study aimed to explore perceived risks of COVID-19 in surgery and to understand how surgical teams innovated to mitigate these risks in the first wave of the pandemic.

Methods:

This qualitative study used semi-structured interviews to examine experiences of surgical teams in the United Kingdom (UK) and internationally. Ethical approval was granted by the University of Bristol Faculty of Health Sciences Research Ethics Committee (reference: 56522). Standards for Reporting Qualitative Research (SRQR) guidelines (17) were followed (Supplementary File 1).

Patient and public involvement

Public and patient involvement was not conducted as part of the current study.

Recruitment and sampling

For peer review only - http://bmjopen.bmj.com/site/about/guidelines.xhtml

Healthcare professionals involved in the management and treatment of patients undergoing surgical procedures were eligible. A key informant sampling approach was initially adopted(18), whereby individuals who were known to the study team were approached. Subsequent participants were identified via snowball sampling(18), whereby interviewees recommended potential participants. Study details were also circulated to members of surgical groups (such as the National Institute for Health Research Surgical MedTech Co-operative and the Royal College of Surgeons of England).

As data collection continued, sampling became increasingly purposeful with a view to achieving a sample of maximum variation, to ensure insights were captured from a range of informants operating in different contexts. We aimed to capture variation in relation to role (including surgeons, theatre nurses and anaesthetists), specialty, years in profession, gender, ethnicity, and geographic location. Capturing an international perspective was important to identify how best practice evolved, particularly in those areas/nations that were 'ahead of the curve'. Participant characteristics were assessed as the study progressed, and interviews with those who were underrepresented were prioritised.

Data collection

Participants provided written consent to take part in the study. Interviews were audio-recorded using an encrypted audio-recorder or videoing conference software. Interviews were conducted by experienced qualitative researchers (DE, MJ, LR, CO, KA, SP, JLD). Discussions were led by a topic guide to ensure that the same core areas were covered, whilst allowing for probing of new issues of importance to participants (Supplementary File 2). Topic guides were adapted as analysis progressed, to enable exploration of emerging findings. Data collection continued until no new key themes were identified (data saturation) as determined by the study team.

Data analysis

Interviews were transcribed verbatim, checked for accuracy and de-identified. Transcripts were imported into NVivo (QSR International, USA), where data were systematically assigned codes by three coders (MJ, LR and CO). Codes were identified inductively using methods derived from grounded theory methodology(19, 20), with new findings systematically compared with existing data. In this way, similarities and differences can be identified and emerging themes refined through the ongoing assimilation of data. Negative cases (i.e. participants whose perspectives/experiences differed from the main body of evidence) were also examined to understand participants' divergent views and to enhance the credibility of the analyses(19). The coders met regularly to compare their

For peer review only - http://bmjopen.bmj.com/site/about/guidelines.xhtml

coding and agree on the main broad categories encapsulating all codes. A subset of the transcripts, batched according to coder, were independently double coded by DE to determine broader consistency in coding approaches.

Descriptive reports were written on key themes (‘Guidance’, ‘Risk’, ‘In-theatre processes’, ‘Organisational’, ‘Service provision’, Personal protective equipment (PPE), ‘Testing’ and ‘Physical and mental impact’). This paper focuses on the findings relating to ‘Risks’, although themes and sub-themes were often intertwined and producing descriptive reports provided an opportunity to demonstrate where there was overlap in key findings. Two coders (CO, MJ) also produced a summary on one theme, to explore any major differences in interpreting codes. As multiple qualitative researchers conducted the interviews, one researcher (DE) read all transcripts and synthesised the findings to ensure consistency in analysis and to enable strategic oversight of the findings. Results, with reference to the raw data, were discussed with members of the wider study team (including academic surgeons) and several research participants who were not part of the study team.

Results:

Nine individuals did not reply to the study invite, said that they were not on the frontline or were unable to find the time for an interview. The final sample of 43 participants included 34 surgeons, five anaesthetists and four individuals in nursing roles. Surgical specialties included general surgery, otolaryngology, neurosurgery, cardiothoracic and ophthalmology. Thirty-one participants were from the UK, and the remaining 12 participants were from Italy (n=3), China (n=1), Spain (n=3), the USA (n=2) and New Zealand (n=3). Participants were mostly male (77%). Interviews were conducted between March-May 2020 and lasted an average of 47 minutes (range=21-116 minutes).

Five sub-themes relating to risk were identified: ‘Facing new and uncertain risks’, ‘The changing context of surgery’, ‘Innovations within high risk specialities, ‘Navigating uncertainty and complex decisions’ and ‘Adapting to PPE-related challenges’. Quotations are provided to support the findings, and these are anonymised to ensure confidentiality.

Facing new and uncertain risks

Participants articulated anxiety about contracting the virus. One individual had personally lost a colleague to COVID-19. Many others described how news of clinical professional fatalities had highlighted the sense of personal risk they and their colleagues were being subjected to.

For peer review only - http://bmjopen.bmj.com/site/about/guidelines.xhtml

HP7: It is just the shock of my life; I had no idea this was coming. I had no idea that walking into the hospital would be dangerous for me... A huge change in my mindset required to deal with this, because it's changed, almost like a war. Like it is normal one day and then within a week, your normal working life is sort of totally changed. Overnight, things changed…it was in the news, we knew it would happen, but then all of a sudden it was here, we were in it.

Whilst they were accustomed to considering risks for patients, interviewees reflected on the shock of how they suddenly faced new risks to their own health. The risk of transmission to family members added a unique dimension to the risks associated with their roles as healthcare professionals.

HP4: And the commonest thing about this whole thing, amongst workers, doctors, nurses, everyone: actually, everyone's worried about catching it. Everyone is terrified of giving it to their family […] I don't want to get it. I really, really don't want to get it. But I'm infinitely more scared about giving it to my wife or my children. And if I catch it, they'll get it. Almost certainly. Because I'll be infected for several days before I'm symptomatic […] We're not used to processing the risk to us… Because these are unfamiliar risks in our practice… The risks that we've always worried about in the past were risks to our patients, or risks to our career. Rather than risks to our health. And as I say, more particularly risks to the health of our families.

The changing context of surgery

COVID-19 had meant that surgical teams had to rapidly assess all aspects of the patient pathway to minimise the potential spread of the virus. For most, the scale of organisational changes was immense and included changes to hospitals, pathways, staff rotas and operating theatres. There was an urgent need to expand the capacity for intensive care, with comprehensive changes that often meant re-purposing operating theatres. There were also considerable discussions about changes to accommodate COVID and non-COVID areas, including separate operating theatres for COVID-19 infected patients and those not infected. These changes required new thinking about patient pathways and processes in operating theatres. Considerable effort went into minimising contact points and the number of people in the operating theatre to avoid potential infection transmission and contamination.

HP5: It is a massive move. Everyone will look at the pathway for every scenario. We are involving everyone and sometimes you are thinking 'Oh yes we have done the pathway now' and then you realise 'oh we missed that one important point. Can we walk through it again?'.

The magnitude of these changes meant that teamworking and communication became even more integral, and many participants described the value of ongoing team discussions with a specific focus

For peer review only - http://bmjopen.bmj.com/site/about/guidelines.xhtml

on COVID-19 related issues. Technology such as video conferencing software or messaging groups had played a huge role to facilitate this.

> *HP3: We were all social distancing at this point and we were having discussions mainly over Microsoft Teams… it's much better now. We started running our cancer MDT on Teams too, that was pretty effective, and we managed to get pretty much the same amount of collaboration or potentially even more because it's quite convenient to be able to dial in from anywhere… I think we'll probably continue to use it in some way all the way into the future hopefully.*

Informants reflected on the challenges of having to adapt as best they could with only limited evidence to guide them. This meant that participants were learning as they went along, and sometimes making mistakes (Table 1). One anaesthetist pointed out the potential implications of *ad hoc* innovations in this unusual time.

> *HP4: One of the most dangerous things in medicine is what's called the MacGyver bias. Which is where people believe that their ability to, to fudge a solution out of something will be much better than the usual way of doing it. I think you always have to be aware that, whilst it's important to adapt and to - in this sort of situation particularly you're having to innovate and change the way you work and change the way you do and change the way you approach things. Always be aware that, if you're the first person to come up with a way of doing it, the chances are that a lot of other people will have thought of that before and decided it's a bloody stupid idea overall.*

For peer review only - http://bmjopen.bmj.com/site/about/guidelines.xhtml

Table 1: Learning lessons the hard way

HP40: We're doing things which may be putting ourselves at more risk than non-risk, because they seem to be sensible at face value, but maybe, are not.. We've been asking patients to wear face masks at the time of surgery… as soon as that happened, the lens we were using for the surgery was steaming up every few minutes. So I thought, "Well, if it's steaming up, it must mean that the patient's breath is coming up the mask and out of the drape that we've put on and into the surgical field." So actually, paradoxically, putting a mask on the patient was probably increasing my exposure in the surgical field, rather than decreasing it. But intuitively, everyone thought that if we put a mask on a patient during surgery, we were reducing our risks… So from a protection perspective, we're still learning, I think. There's no doubt that we will be better prepared for the next wave that is being predicted.
HP4: We turned the top floor into an intensive care unit. And when that started filling up, we realised it was just a really bad idea. Because, although it's a nice idea having patients isolated in separate rooms, if a patient is in a separate room, they need one nurse to look after them in each room. And so, it's very nurse heavy. Whereas, actually, once you start getting a lot of patients, what you need is to be able to cohort the patients. So, 3 or 4 in a bay. And you can have 1 or 2 non-skilled nurses - you know, not ITU-skilled nurses - in there being supervised by 1 ITU nurse. So, you can dilute your skills… The other thing with that plan, the idea was, as our number of patients increased, we could keep them all in the same block and we would just expand intensive care down from the top floor, to the floor below, to the floor below that but unfortunately … there was only an adequate back-up power supply to the top floor. And the next 2 floors below had nowhere near enough back-up power. So, if the mains power went down, we'd kill everyone on the ward instantly. So, we've had to abandon that completely and decant an entire intensive care unit to somewhere else and open a new one up. And build another intensive care unit. Because one of things no-one had thought to check was, actually, was there enough power there.
HP37: So, if you have, for instance, three intubated patients connected to the ventilator, all the ventilators have a lot of filters, with a high-quality filtering system… it was a challenge, because the filters, you have to remove them and change them every day… because otherwise they can clot and they can increase the resistance in the ventilator.
HP4: Our plan was always going to be we would collect the patients take them to intensive care, anaesthetise them there and do everything there. But we very swiftly realised that that actually meant a trip with a potentially very unstable patient, through the hospital, not in a controlled situation. We risk contaminating what may be clean bits of the hospital. And so then we actually moved to completely just doing everything on site. So, we essentially take intensive care to the patients. And that saves a lot of time. We think made the process much safer for the patients. And made the process safe for everyone else in the hospital as well. Because people who were infected were only then being moved through the hospital a minimal amount of time, to decrease contamination risk.

For peer review only

Innovations within high risk specialities

There was agreement that healthcare professionals who participated in procedures within the head and neck region were at increased risk of infection. For instance, anaesthetists were at high risk due to aerosolisation when intubating and extubating patients due to direct access or direct instrumentation of the airway. There were many strategies adopted in these contexts (Table 2), including changes to where patients were intubated and extubated to minimise points of patient transfer, methods to reduce aerosolisation during intubating and extubating (such as having drapes or sheets), pausing to allow aerosols to be dissipated before additional people came into theatre, or avoiding intubation all together by changing the type of anaesthesia from general to local.

> *HP40: One of the innovations that has happened as a direct result of COVID is that surgical procedures that, up until now, we would have said were general anaesthetic procedures have been successfully*

For peer review only - http://bmjopen.bmj.com/site/about/guidelines.xhtml

converted to local procedures… We would never have contemplated doing an enucleation – removing the eye – under a local anaesthetic before. Now, we have successfully done that.

Table 2: Modifications to practice for intubating patients

Example	Quotation
**Changes to where the patient is intubated**	*HP28: The anaesthetic is being conducted in the theatre whereas normally it would be done in the anaesthetic room in the UK … It just means that they are not moving between lots of different places as well, so they try to minimise the number of points that the patients will stop at.*
**Changes to where patient extubated**	*HP4: Now we would actually wake the patient up in theatre and have them awake in theatre before they went out, where previously, patients would routinely be taken to the recovery area mostly asleep... In the past, where you might have a patient waking up with an airway in, coughing it out, coughing and spluttering a bit. But actually if they do all that whilst we're in the theatre environment, which is already contaminated, and we're all in protective kit.*
**Reducing aerosolisation during intubation**	*HP34: If you put a plastic sheet over the head of the patient that reduces much of the aspiration, so the spit, coming out and then the aerosolisation which causes spit will sit on that plastic more than hit you in the face or the neck or anything like that. Those are good things that came out and you can actually see it, the one I saw, you saw all of the spit on the patient, when you extubate, the spit went all over the patient, if you didn't have that plastic sheet, that would be, because your neck is still exposed and all of that.*
**Pausing to allow aerosols to be dissipated**	*HP17: There's a delay between the patient being put to sleep and the operation being started, just to allow the air to be changed in theatres. The rationale behind that being that when someone's put to sleep and they've got a breathing tube put down, the virus might be kind of put into the air, they might be floating around. Operating rooms have got incredibly high air flow, the air is changed-, it depends on your particular operating theatre but each individual hospital will know how long it takes to cycle the air out of their theatre, and then they can do a bit of maths to see how long it takes for the air in there to be entirely fresh. It's normally about 20, 25 minutes.*

Media coverage showing deaths of ENT surgeons appeared to have impacted on the surgical community's perception of risk for this group, and many described how it made them scrutinise their own practice. Some of the ways that tracheostomies, for example, were adapted to try to mitigate this risk are outlined in Table 3. This included tracheostomies being performed only by the most experienced surgeons to maximise efficiency and safety as much as possible, having minimal people involved, pausing the ventilator during intubation, and changing the location of the procedure to minimise patient transfer.

HP10: Basically the highest concentration of virus in an ill Covid patient is in anything that comes in and out of their mouth and nose, if they've got Covid contains the highest concentration of viral particles with the greatest risk of cross infection to somebody looking after that patient. Very sadly, the first Doctor to die in the UK was an ENT surgeon and they spend their entire lives examining peoples' upper aerodigestive tract and therefore they were at particularly high risk.

For peer review only - http://bmjopen.bmj.com/site/about/guidelines.xhtml

Table 3: Modifications to forming a tracheostomy

Example	Quotation
**Being performed only by a small team of experts**	*HP43: Early on, I was interested in working [on tracheotomies] and a surgeon was interested in it as well. Our personalities work reasonably well together and we started, from the surgical side at least, combining forces and trying to figure out a way to do this safely… We weren't exactly sure which patients were going to make the most sense. We didn't know who exactly would benefit from it, but there was little question there were going to be some people … We go into the room, we wheel in all our stuff. We don't even talk that much anymore. Teamwork is very crucial… I think it has been important, both from an operational standpoint, but also from a mental support and enthusiasm standpoint. I think, if one of us was doing this alone, without the other, I think it would be a lot more difficult. I wouldn't say it's undoable on your own completely, but it's just crazy to try to do that. You have to ask for help.*
**Pausing the ventilator**	*HP41: I think a tracheotomy is more risk… Before we cut open, cut open the trachea, we make the ventilator pause for a second. Then first we pause the ventilator, then we cut, and then we insert the intubation quickly, and we restart the ventilator … If the ventilator is open, then there are a lot of aerosols… Secretion maybe comes out from the lungs, from the bronchia. So if we pause the ventilator, maybe they can decrease the secretions.*
**Location of tracheotomy**	*HP41: All the tracheotomy is at the bedside. So it's including a resident, to help at the head side of the patient, and two surgeons. One is at the bedside, to do the surgery. And one or two nurses help to deliver some things to help the surgery. Now, the unit is the ICU unit. There's one person per room.*

Navigating uncertainty and complex decisions

Participants described how new national and local guidelines had stated that AGPs such as laparoscopic and endoscopic approaches subjected staff to elevated risk. However, many reflected that there had been some uncertainty as to precisely what constituted an AGP within their surgical teams. There were also conflicting views as to whether, in line with guidance, alternative techniques should be adopted.

> *HP13: It's really uncertain. We're bombarded by information from national societies, international societies, there's webinars left right and centre, and I've certainly taken part in a fair few of those to try and understand peoples' perspectives. And we've got that sort of headline idea that surgeons are at risk from aerosolised procedures, but we don't really have any knowledge of what an aerosolising procedure is.*

When contemplating whether to proceed with AGPs, teams weighed up many factors, including the potential risks to the patient, existing evidence (or lack thereof), and what colleagues or those in the wider surgical community were doing. Taken together, informants' accounts suggested that the issue of AGPs created conflicts between their clinical roles where they had to weigh up what was best for their patients whilst contemplating their own safety.

For peer review only - http://bmjopen.bmj.com/site/about/guidelines.xhtml

> *HP19: It's not all about saving the physician and the team, it's about everybody in the process and understanding that these are really really, difficult decisions… They're not black and white, they're grey… It's about protecting surgeons, it's not about protecting patients... I'm not sure there's been another example where it's been so clear that effectively national guidance has come out to say you put yourself ahead of the patient care, which is how I interpret the guidance that came out.*

As the quotes below highlight, informants were conflicted as to how comfortable they felt about putting their own safety ahead of the patient's.

> *HP28: We have to accept risks to us because the patient needs an operation far more than we need to be protected… You look at the newspapers and the clinicians and the nursing staff and the porter staff that have all fallen foul of this, people are going to continue to work, there's an inherent dedication to achieving a safe outcome for a patient if that necessarily places you at risk as well.*

> *HP9: Although you want to do everything for your patient, your own safety and the safety of your team has to come first because if you have a whole team who is not properly protected and everybody gets infected you have taken out a team or an individual of that team for a period of time, so they can't help with the future patients.*

When proceeding with AGPs, participants described adopting the highest level of PPE and implementing strategies and innovations to try to minimise the spread of the virus during aerosolisation (Table 4). This included utilising filters on each port to stop gas escaping and using drapes to provide a 'barrier' to aerosols passing through the air.

Table 4: Modifications to AGPs

Examples	Quotation
**Wearing full PPE**	*HP28: We would use keyhole surgery and we use various adaptations for the keyhole …we did everything with PPE at the time we saw the patient, full PPE in theatre […] We went against guidance and used keyhole surgery because we felt that there was no evidence to suggest that it's any more risky.*
**Use of smoke-filter evaporators**	*HP21: We use filters to avoid aerosols, every port is connected to a filter so no aerosol comes through the port... We fix the port to the skin to avoid accidental removal, apply a stitch to the port and fix it to the skin, so the chances of the port being accidentally removed is really decreased.*
**Plastic sheets**	*HP12: We cover the head and the drill with this plastic sheet that aerosols are not going into the air, and not going everywhere, to protect the environment and to protect the surgeons.*
**Drapes**	*HP18: We're using some simple things like some plastic drapes over the top of the really aerosol generating stuff that we're doing, making some holes in those drapes so that we can still use our normal instruments.*
**Adopting a combination of**	*HP20: Tracheostomies were done percutaneously … They have done some modification, doing some combination between open and percutaneous*

For peer review only - http://bmjopen.bmj.com/site/about/guidelines.xhtml

**approaches**	*tracheostomy…to minimise as much as possible the risk of leaking aerosol in the operative field to the operative team.*

Adapting to PPE-related challenges

At the time of interviews, most participants felt that the PPE provided was sufficient, although some interviewees expressed their concerns about not having access to adequate PPE and described having to outsource their own or 'pushing' their hospitals to provide more or fuller PPE. Moreover, several informants reflected on the news coverage that there had been limited PPE available to hospital staff, and expressed concerns that this would happen to them as the pandemic progressed. As guidance emerged stating that healthcare professionals should not put themselves at risk if they did not have sufficient PPE, many were again forced to consider whether to put their own safety before their patients':

> *HP4: Bottom line is, I have a wife and children […] I am not going to put my family at risk by doing something without the right kit. Sorry. That's the way it is. If I haven't got the equipment to do it, I'm not doing […] There's national guidelines on what we should wear for each situation. And if we haven't got it, I'm not doing it.*

Some participants also expressed concern as to whether their PPE was fitted correctly, and teams developed local strategies to facilitate optimal PPE donning/doffing. This included creating visual aids (checklists, signs/posters in theatre), having 'buddy systems' and engaging in team simulations to practice and contemplate different donning/doffing scenarios. Most informants reported that wearing PPE was hot, distracting and uncomfortable, although there was a clear sense that the discomfort was a necessary compromise for sufficient protection.

> *HP3: I am just constantly thinking, have I done this right? Am I putting this on in the right way? Is my mask fitting properly? I just don't know, and then the real feeling of… I don't want to, I really don't want this on my face during this procedure, and am I doing the right thing. And I think one of the things for me is that I don't really feel like we have had very good instructions on how to do these things. So, one of the things that hit me this morning was, nobody really seems to know, nobody seems to be in control, or what's going to happen and these are the processes, this is where you put your stuff. So when you are putting it on and taking it off etc etc, it is a bit chaotic and that makes me really nervous… I hope I got it right, but I don't know.*

Wearing PPE in the operating room also created practical problems that had the potential to impact performance during surgery. The time taken to don PPE was considered problematic in emergency circumstances. One strategy for enhancing efficiency was to pre-pack PPE in 'grab and go' bags to enable staff to proceed more swiftly to theatre. PPE made it significantly harder to communicate

For peer review only - http://bmjopen.bmj.com/site/about/guidelines.xhtml

between staff in theatre and between staff positioned in 'clean' and 'dirty' areas. Staff were also unable to obtain additional surgical equipment whilst in the operating theatre. Consequently, many participants described strategies to facilitate communication (Table 5), including changing the roles of 'runners' and using two-way radios ('walkie talkies') and whiteboards.

Table 5: Problems caused by PPE and potential strategies to overcome these issues

Issue caused by PPE	Quotation	Potential solution	Quotation
**Time taken to don PPE**	*HP19: It's made the operations much longer, by the time you get the kit on.*	**'Grab bags'**	*HP4: We've set up a fantastic production line of grab bags. So, everything we need is in one of a series of grab bags. […] We're going off to intubate someone, so we want that bag and that bag. We're going to put lines in them, so we want that bag.*
**Difficulty determining who is who**	*HP3: You can't take name badges in.*	**Name and roles written on PPE**	*HP11: People do write their names on their gowns or aprons… The problem is you don't know what their role is. They should actually write, 'ITU Nurse'.*
**Difficulty communicating whilst wearing full PPE**	*HP11: I could barely hear anybody. It was really hard.*	**Using equipment to facilitate communication**	*HP34: Vocera is a communications system that you can have clipped on you or if you're in the scrub role you can have it on the side and you can activate it by clicking it. With the N95 mask you don't hear people. If you have Vocera then you can speak and people can hear.*
**PPE fogging up glasses**	*HP19: When I was wearing my spectacles when I was breathing out the air was misting up my spectacles.*	**Masks over glasses**	*HP20: We have been working on the last week on a modification. You have seen some reports in different countries about the use of a snorkel mask.. and it seems to work much better, without fogging.*
**Needing additional equipment**	*HP3: Normally if we need something extra during the operation, someone just walks out of theatre, get its and then comes back in again, but we are not allowed to do because there are clean areas and dirty areas.*	**Equipment to aid communication with 'runners'**	*HP8: Well I brought some walky-talkies which are quite good which help with communicating from inside to outside of the room. So that is one problem. They are quite useful… the runner outside will have one and we have one in a plastic bag in the theatre. I actually bought them on eBay and everyone wants more now so I am buying some more which should arrive any day…. Shouting through the door or holding up signs is difficult. These work quite well actually.*<br>*HP3: One of the anaesthetists had the idea of having some Bluetooth speakers, so one in the clean area and one in the dirty area.*

For peer review only - http://bmjopen.bmj.com/site/about/guidelines.xhtml

**Long periods wearing PPE**	*HP34: We realised people can't do nine hours in a theatre with PPE on, wearing the N95 puts incredible pressure on people's faces.*	**Changes in staffing**	*HP35: We have to do a team change, so we changed how we did things there as well. We started looking at team changes and how we would manage those, we'd send people home, knowing that in 4 hours, they'd be coming in to do a team change.*

For some, PPE affected how operations were performed. Surgeons described, for example, how wearing two sets of gloves affected their dexterity and how using a microscope was difficult whilst wearing a visor. These participants described removing their PPE to complete specific tasks. The issue of surgeons having to weigh up being fully protected by PPE and their ability to operate once again demonstrates the tension between the safety of patients and healthcare professionals.

> *HP16: The problem surgically with wearing the visors are that the visors sticks out from your face, so I desterilise the instrument on the visor and I desterilise my gloves and the visor so then at the end of the procedure I ended up just having to take the visor off because it was a sterility risk for the procedure I was trying to perform. Trying to use the operating microscope with the visor or with googles on outside of my glasses is just not possible. So at that point, I have areas of my face exposed while I am looking down the operating microscope puttering away at a tumour over creating aerosols whilst not fully covered and I can't be fully covered, else I can't do the surgery if I am.*

Discussion:

This study captures how surgical teams were suddenly faced with new and unfamiliar risks due to COVID-19. We report how they made sense of and adapted to these risks as the pandemic unfolded. Informants described how there was a plethora of evolving – and sometimes contradictory – local and national recommendations about providing surgical care safely during the pandemic. Our study highlights that guidelines were not a panacea for surgical practice, and at times, there were tensions between clinical roles and personal safety. Perceptions of risk were complex and context-dependent. Surgical teams sought to mitigate the risks by modifying their current practice with multiple strategies and innovations to deliver patient care that they felt comfortable with. Nonetheless, uncertainties remained about optimal strategies to minimise risks. Although the epidemiology of COVID-19 has been well reported, there is little robust evidence regarding safe and best surgical practice (21). Our study supports an urgent need for high-quality multicentre research (13), particularly as a second wave is beginning and the pandemic continues to disrupt health services around the world. It has been argued that with the constraints of time and resource, clinical practice must be driven by a pragmatic and novel approach to collate global evidence and to inform evidence-based practice (21).

For peer review only

This study has also highlighted that COVID-19 has had a significant impact on healthcare professionals. This includes experiences of fear and anxiety about personal safety, the challenges of making sense of multiple guidelines that changed frequently, an evolving workload and wearing more PPE. This is supported by findings from interviews with fourteen surgeons from a hospital in Ireland, which reported how COVID-19 had fundamentally impacted clinical roles and had consequences on wellbeing (22). It has been argued that the surgical community need to be provided with support to limit the inevitable psychological burden and risk of burnout (23-26). Shanafelt and colleagues also emphasise that visible leadership is crucial during this turbulent time, and that leaders will need to establish innovative ways to engage with their teams to fully listen to and understand the sources of concern, and work with them to develop approaches that mitigate concerns (27).

The main strength of this research is the use of qualitative methodology to provide important and rich insights into perceived risks in surgery in the context of COVID-19. Initially, individuals who were known to the study team were approached to consider participation and subsequent participants were identified via snowball sampling. It is therefore possible that the findings may not be representative of all healthcare professionals. The researchers in this study were known to have a particular interest in surgical innovation and responses may have also been influenced by participants' perceptions of what might constitute socially desirable answers, particularly given the emotive nature of some of the topics discussed in the interviews (e.g. resource constraint, balancing risks to patients versus risks to themselves). However, we were able to capture a range of perspectives from surgical teams to explore variability between centres and countries and explore negative cases that contradicted with emerging findings to understand participants' divergent views and to enhance the credibility of the results(19). Measures were undertaken to ensure rigour of the data in ensuring findings reflected the meaningful impact of COVID-19 on surgical teams. Having several trained qualitative methodologists conducting the interviews and analysis provided an opportunity to gain multiple perspectives on the study findings. One individual double-coded a proportion of transcripts to ensure consistency in analysis and reviewed each transcript to enable strategic overview of the study findings. Emerging themes, with reference to the raw data, were discussed with wider members of the study team (including academic surgeons), as well as research participants who were not part of the study team, to check the credibility of the findings (28).

It is important to note that interviews were conducted between March and May 2020. Whilst we were able to capture initial responses as the pandemic unfolded in its first wave, future research is

For peer review only - http://bmjopen.bmj.com/site/about/guidelines.xhtml
For peer review only

warranted to continue to explore how teams continue to adapt. It must also be noted that participants were from the UK, Italy, China, Spain, the USA and New Zealand; all developed countries. Risk mitigation in surgery may look very different in low and middle income countries with fewer resources, different healthcare infrastructures and more recent experience of epidemics. Capturing a wider range of international perspectives would inform the wider discourse on optimal practices for surgical procedures during the pandemic.

In summary, our study provides insights into how surgical teams had to rapidly modify their practice with a wide range of innovations to mitigate the risk of COVID-19. Communication and teamwork played an integral role in how teams adapted. Whilst an abundance of guidance was available, perceptions of risks were complex and context dependent. Taken together, this study highlights that the context of surgery has changed dramatically and that there are many uncertainties about best practice. As the COVID-19 pandemic continues to unfold, it is imperative that future research continues to explore how teams adapt and that surgical practice is based upon high-quality evidence that optimises the safety of both patients and healthcare professionals.

Author contributions:

DE was the project lead and developed the study protocol, with all authors reviewing and providing comments on subsequent versions. DE, CO, LR, MJ, KNLA, SP and JLD conducted interviews. DE, CO, LR, MJ and JLD analysed the data. JMB, MB, NSB, PH, DJ, DE, MJ, LR and JLD identified potential study participants. DE drafted the initial version of the manuscript, with critical revisions from JLD, JMB and LR. JMB, MB, NB, DJ and PH conceptualised the study design. All authors commented on the manuscript and gave approval for the manuscript to be submitted. The NIHR Bristol Biomedical Research Centre Surgical Innovation theme is directed by JMB (theme lead).

Disclosure: The authors declare no conflict of interest.

Acknowledgements: We would like to thank Rosetrees Trust for funding this study, and each participant for taking the time to share their experiences with us. We are also grateful to Murat Akkulak, Steven Beech, Becky Carthy, Jenny Russe, Jane Collingwood, Chris Pawsey and Tom Steart-Feilding for providing help and support with the study.

For peer review only - http://bmjopen.bmj.com/site/about/guidelines.xhtml

References

1. Organization WH. Coronavirus disease 2019 (COVID-19): situation report, 72. 2020.
2. Tay JK, Khoo ML-C, Loh WSJJOH, Surgery N. Surgical considerations for tracheostomy during the COVID-19 pandemic: lessons learned from the severe acute respiratory syndrome outbreak. 2020.
3. Wang J, Zhou M, Liu FJTJoHI. Reasons for healthcare workers becoming infected with novel coronavirus disease 2019 (COVID-19) in China. 2020.
4. Chang D, Xu H, Rebaza A, Sharma L, Cruz CSDJTLRM. Protecting health-care workers from subclinical coronavirus infection. 2020;8(3):e13.
5. Givi B, Schiff BA, Chinn SB, Clayburgh D, Iyer NG, Jalisi S, et al. Safety Recommendations for Evaluation and Surgery of the Head and Neck During the COVID-19 Pandemic. 2020.
6. Brindle M, Gawande AJAoS. Managing COVID-19 in surgical systems. 2020.
7. Collaborative C. Global guidance for surgical care during the COVID-19 pandemic.n/a(n/a).
8. Mayol J, Fernández Pérez C. Elective surgery after the pandemic: waves beyond the horizon. The British journal of surgery. 2020:10.1002/bjs.11688.
9. Wong J, Goh QY, Tan Z, Lie SA, Tay YC, Ng SY, et al. Preparing for a COVID-19 pandemic: a review of operating room outbreak response measures in a large tertiary hospital in Singapore. 2020:1-14.
10. Thornton JJb. Clinicians are leading service reconfiguration to cope with covid-19. 2020;369.
11. Hutchison K, Rogers W, Eyers A, Lotz M. Getting Clearer about surgical innovation: a new definition and a new tool to support responsible practice. Ann Surg. 2015;262(6):949-54.
12. Ti LK, Ang LS, Foong TW, Ng BSWJCJoAJcda. What we do when a COVID-19 patient needs an operation: operating room preparation and guidance. 2020:1-3.
13. Collaborative CJTBjos. Global guidance for surgical care during the COVID-19 pandemic. 2020.
14. Søreide K, Hallet J, Matthews JB, Schnitzbauer AA, Line PD, Lai PBS, et al. Immediate and long-term impact of the COVID-19 pandemic on delivery of surgical services. The British journal of surgery. 2020:10.1002/bjs.11670.
15. Teti M, Schatz E, Liebenberg L. Methods in the Time of COVID-19: The Vital Role of Qualitative Inquiries. SAGE Publications Sage CA: Los Angeles, CA; 2020.
16. Leach M, Parker M, MacGregor H, Wilkinson AJIoDS. COVID-19—A social phenomenon requiring diverse expertise. 2020.
17. O'Brien BC, Harris IB, Beckman TJ, Reed DA, Cook DAJAM. Standards for reporting qualitative research: a synthesis of recommendations. 2014;89(9):1245-51.
18. Dempster M. A Research Guide for Health and Clinical Psychology: Palgrave Macmillan; 2011.
19. Glaser BG, Strauss AL. The Discovery of Grounded Theory. Aldine Transaction: USA; 1967.
20. Seale C, Silverman DJTEJoPH. Ensuring rigour in qualitative research. 1997;7(4):379-84.
21. Welsh Surgical Research Initiative C. Recommended operating room practice during the COVID-19 pandemic: systematic review. BJS Open. 2020:10.1002/bjs5.50304.
22. Whelehan DF, Connelly TM, Ridgway PFJTS. COVID-19 and surgery: A thematic analysis of unintended consequences on performance, practice and surgical training. 2020.
23. Spinelli A, Pellino G. COVID-19 pandemic: perspectives on an unfolding crisis. The British journal of surgery. 2020;107(7):785-7.
24. de Pablo GS, Serrano JV, Catalan A, Arango C, Moreno C, Ferre F, et al. Impact of coronavirus syndromes on physical and mental health of health care workers: Systematic review and meta-analysis. 2020.
25. Zerbini G, Ebigbo A, Reicherts P, Kunz M, Messman HJGGMS. Psychosocial burden of healthcare professionals in times of COVID-19–a survey conducted at the University Hospital Augsburg. 2020;18.
26. Dzau VJ, Kirch D, Nasca TJNEJoM. Preventing a parallel pandemic—a national strategy to protect clinicians' well-being. 2020.
27. Shanafelt T, Ripp J, Trockel MJJ. Understanding and addressing sources of anxiety among health care professionals during the COVID-19 pandemic. 2020;323(21):2133-4.
28. Guba EG, Lincoln YSJHoqr. Competing paradigms in qualitative research. 1994;2(163-194):105.

For peer review only

Reporting checklist for qualitative study.

Based on the SRQR guidelines.

Instructions to authors

Complete this checklist by entering the page numbers from your manuscript where readers will find each of the items listed below.

Your article may not currently address all the items on the checklist. Please modify your text to include the missing information. If you are certain that an item does not apply, please write "n/a" and provide a short explanation.

Upload your completed checklist as an extra file when you submit to a journal.

In your methods section, say that you used the SRQRreporting guidelines, and cite them as:

O'Brien BC, Harris IB, Beckman TJ, Reed DA, Cook DA. Standards for reporting qualitative research: a synthesis of recommendations. Acad Med. 2014;89(9):1245-1251.

		Reporting Item	Page Number
**Title**			
	#1	Concise description of the nature and topic of the study identifying the study as qualitative or indicating the approach (e.g. ethnography, grounded theory) or data collection methods (e.g. interview, focus group) is recommended	1
**Abstract**			
	#2	Summary of the key elements of the study using the abstract format of the intended publication; typically includes background, purpose, methods, results and conclusions	3
**Introduction**			
Problem formulation	#3	Description and significance of the problem / phenomenon studied: review of relevant theory and empirical work; problem statement	5

For peer review only

Purpose or research question	#4	Purpose of the study and specific objectives or questions	5
**Methods**			
Qualitative approach and research paradigm	#5	Qualitative approach (e.g. ethnography, grounded theory, case study, phenomenolgy, narrative research) and guiding theory if appropriate; identifying the research paradigm (e.g. postpositivist, constructivist / interpretivist) is also recommended; rationale. The rationale should briefly discuss the justification for choosing that theory, approach, method or technique rather than other options available; the assumptions and limitations implicit in those choices and how those choices influence study conclusions and transferability. As appropriate the rationale for several items might be discussed together.	5
Researcher characteristics and reflexivity	#6	Researchers' characteristics that may influence the research, including personal attributes, qualifications / experience, relationship with participants, assumptions and / or presuppositions; potential or actual interaction between researchers' characteristics and the research questions, approach, methods, results and / or transferability	5-6, 14-15
Context	#7	Setting / site and salient contextual factors; rationale	5-6
Sampling strategy	#8	How and why research participants, documents, or events were selected; criteria for deciding when no further sampling was necessary (e.g. sampling saturation); rationale	5-6
Ethical issues pertaining to human subjects	#9	Documentation of approval by an appropriate ethics review board and participant consent, or explanation for lack thereof; other confidentiality and data security issues	5
Data collection methods	#10	Types of data collected; details of data collection procedures including (as appropriate) start and stop	6

For peer review only

		dates of data collection and analysis, iterative process, triangulation of sources / methods, and modification of procedures in response to evolving study findings; rationale	
Data collection instruments and technologies	#11	Description of instruments (e.g. interview guides, questionnaires) and devices (e.g. audio recorders) used for data collection; if / how the instruments(s) changed over the course of the study	6
Units of study	#12	Number and relevant characteristics of participants, documents, or events included in the study; level of participation (could be reported in results)	7
Data processing	#13	Methods for processing data prior to and during analysis, including transcription, data entry, data management and security, verification of data integrity, data coding, and anonymisation / deidentification of excerpts	6-7
Data analysis	#14	Process by which inferences, themes, etc. were identified and developed, including the researchers involved in data analysis; usually references a specific paradigm or approach; rationale	6-7
Techniques to enhance trustworthiness	#15	Techniques to enhance trustworthiness and credibility of data analysis (e.g. member checking, audit trail, triangulation); rationale	6-7
**Results/findings**			
Syntheses and interpretation	#16	Main findings (e.g. interpretations, inferences, and themes); might include development of a theory or model, or integration with prior research or theory	7-13
Links to empirical data	#17	Evidence (e.g. quotes, field notes, text excerpts, photographs) to substantiate analytic findings	7-13
**Discussion**			
Intergration with prior work, implications,	#18	Short summary of main findings; explanation of how findings and conclusions connect to, support, elaborate on, or challenge conclusions of earlier	13

For peer review only

transferability and contribution(s) to the field		scholarship; discussion of scope of application / generalizability; identification of unique contributions(s) to scholarship in a discipline or field	
Limitations	#19	Trustworthiness and limitations of findings	14-15
**Other**			
Conflicts of interest	#20	Potential sources of influence of perceived influence on study conduct and conclusions; how these were managed	14-15
Funding	#21	Sources of funding and other support; role of funders in data collection, interpretation and reporting	1-2

The SRQR checklist is distributed with permission of Wolters Kluwer © 2014 by the Association of American Medical Colleges. This checklist was completed on 12. February 2020 using https://www.goodreports.org/, a tool made by the EQUATOR Network in collaboration with Penelope.ai

For peer review only

Understanding risks, mitigation and innovations for Surgery in a COVID19 world: Topic guide

Introduction

- Thank you for taking the time to talk. Know there is so much going on at the moment and will try not to take too long.
- You can stop the interview at anytime.
- Purpose of interview:
    - We know that COVID19 presents new risks in theatre and to operating.
    - Across the world all sorts of ideas are emerging to mitigate the risk and experience is accumulating.
    - The purpose of our interview study is to listen to your **views and to understand your experiences and any solutions you have found to these challenges**.
    - We find that personal views of complex situations may yield information not accessed by standard methods. We're hoping to identify useful ideas and we intend to share good practice from this study rapidly in real time (anonymised data) to help others.
- Am quali researcher at UoB (not HP – apologies if some questions seem silly).
- Everything confidential, quotes anonymous.

Check recording

- Will send/have sent written consent form but can I just confirm you're happy to take part in the interview and for me to record it?

For peer review only

<u>**Impact of COVID19**</u>

How are you? How things have been in hospital since the pandemic?

- Can you talk to me about how Covid19 has affected surgery? *(Consider both elective and emergency procedures).*
- Which procedures are considered 'essential' during this time? *How judged? Who/how decisions made?*
- In your opinion what are the key risks in surgery due to Covid19? How identified these risks? *(training, guidance, experience)*
- Which procedures within your specialty are at a higher risk during this time? Why?

Can you describe a case where COVID19 affected what happens in the operating theatre?

- In Covid confirmed patients, those suspected, or all?
- Which staff at risk?

<u>**PPE**</u>

I'm still getting to grips with what constitutes as PPE. In your own words, can you talk me through how PPE has been used during Covid19? (ie – masks, eyewear, full hazmat suits)

- Thoughts on these in terms of:
    - *Practicalities?*
    - *Comfort?*
    - *Communication?*
    - *Whether training provided/needed?*
    - *Availability?*
    - *Does the equipment provide sufficient mitigation to risks of spread? If not, why not?*
    - *What would you class as minimal/optimal PPE?*
    - *Are there different examples for different procedures?*
    - *Necessary for confirmed Covid19 patients, suspected, or all patients, at this time?*

<u>**Role of team**</u>

How has COVID19 affected the theatre team?

- Are staff self-isolating if COVID19 suspected? Are staff tested?
- The RCS Guidance refers to 'minimal theatre staff'. Has Covid19 affected who is present? *How do you define 'minimal? Who do you think SHOULD be there (essential/ desirable)*
- Has COVID affected theatre time? *Pre/post-op prep?*

<u>**Considering other factors**</u>

What else is important? Is there anything else do we need to know or info we should we share with others? Consider:

- Surgical approaches that may be safer/riskier? *(e.g. laparoscopic, endoscopic, open – and implications of changing techniques)*
- Pre-surgical prep?
- Handwashing?

- Location of surgery *(e.g. anteroom)*
- Affected how anaesthesia used?

Given that so much has changed because of Covid19, how will surgery be in the future?

- How to re-start surgery that has stopped?

Ending the interview

- Summarise key info – what are key lessons learned regarding safety in surgery? Anything else important?
- Thank for time
- Request additional people to interview

For peer review only

BMJ Open

"Overnight, things changed. Suddenly, we were in it": A qualitative study exploring how surgical teams mitigated risks of COVID-19

Journal:	*BMJ Open*
Manuscript ID	bmjopen-2020-046662.R1
Article Type:	Original research
Date Submitted by the Author:	20-Apr-2021
Complete List of Authors:	Elliott, Daisy; University of Bristol, Population Health Sciences<br>Ochieng, Cynthia; University of Bristol, Population Health Sciences<br>Jepson, Marcus; Population Health Sciences<br>Blencowe, Natalie; University of Bristol, Population Health Sciences<br>Avery, Kerry; University of Bristol, Population Health Sciences<br>Paramasivan, Sangeetha ; University of Bristol, Population Health Sciences<br>Cousins, Sian; University of Bristol, Population Health Sciences<br>Skilton, Anni; University of Bristol<br>Hutchinson, Peter; University of Cambridge, Academic Neurosurgery<br>Jayne, David; Leeds Teaching Hospitals NHS Trust<br>Birchall, Martin; University College London Ear Institute<br>Blazeby, Jane; University of Bristol, Population Health Sciences<br>Donovan, Jenny; University of Bristol, Population Health Sciences<br>Rooshenas, Leila; University of Bristol, Population Health Sciences
**Primary Subject Heading**:	Qualitative research
Secondary Subject Heading:	Health services research, Surgery
Keywords:	QUALITATIVE RESEARCH, SURGERY, COVID-19

SCHOLARONE™
Manuscripts

I, the Submitting Author has the right to grant and does grant on behalf of all authors of the Work (as defined in the below author licence), an exclusive licence and/or a non-exclusive licence for contributions from authors who are: i) UK Crown employees; ii) where BMJ has agreed a CC-BY licence shall apply, and/or iii) in accordance with the terms applicable for US Federal Government officers or employees acting as part of their official duties; on a worldwide, perpetual, irrevocable, royalty-free basis to BMJ Publishing Group Ltd ("BMJ") its licensees and where the relevant Journal is co-owned by BMJ to the co-owners of the Journal, to publish the Work in this journal and any other BMJ products and to exploit all rights, as set out in our licence.

The Submitting Author accepts and understands that any supply made under these terms is made by BMJ to the Submitting Author unless you are acting as an employee on behalf of your employer or a postgraduate student of an affiliated institution which is paying any applicable article publishing charge ("APC") for Open Access articles. Where the Submitting Author wishes to make the Work available on an Open Access basis (and intends to pay the relevant APC), the terms of reuse of such Open Access shall be governed by a Creative Commons licence – details of these licences and which Creative Commons licence will apply to this Work are set out in our licence referred to above.

Other than as permitted in any relevant BMJ Author's Self Archiving Policies, I confirm this Work has not been accepted for publication elsewhere, is not being considered for publication elsewhere and does not duplicate material already published. I confirm all authors consent to publication of this Work and authorise the granting of this licence.

For peer review only

"Overnight, things changed. Suddenly, we were in it": A qualitative study exploring how surgical teams mitigated risks of COVID-19

Authors:

Daisy Elliott PhD[1], Cynthia Ochieng PhD[1], Marcus Jepson PhD[2], Natalie S Blencowe PhD[1,3], Kerry NL Avery PhD[1], Sangeetha Paramasivan PhD[2], Sian Cousins PhD[1], Anni Skilton MSc[1], Peter Hutchinson PhD FRCS[4], David Jayne MD[5], Martin Birchall MD[6], Jane M Blazeby PhD FRCS [1,3], Jenny L Donovan PhD[2], Leila Rooshenas PhD[2]

Affiliations:

[1] National Institute for Health Research Bristol Biomedical Research Centre, Surgical Innovation Theme, Centre for Surgical Research, University of Bristol, 39 Whatley Road, Clifton, Bristol, UK, BS8 2PS

[2] Population Health Sciences, Bristol Medical School, University of Bristol, 39 Whatley Road, Clifton, Bristol, UK, BS8 2PS

[3] University Hospitals Bristol NHS Foundation Trust, Bristol, UK, BS2 8HW

[4] Division of Neurosurgery, Department of Clinical Neuroscience, University of Cambridge, Cambridge CB2 2QQ

[5] Leeds Institute of Medical Research, Level 7 Clinical Sciences Building, St. James's University Hospital, Leeds, LS9 7TF

[6]Ear Institute, Faculty of Brain Sciences, UCL, London

Correspondence to: Daisy Elliott [daisy.elliott@bristol.ac.uk]

Running title: Mitigating COVID-19 risks in surgery

Keywords: Surgery; COVID-19, Risks; Qualitative

Submission type: Original article

Funding statement: This study was funded by the Royal College of Surgeons (RCS Covid Research Group) and Rosetrees Trust, with support from the NIHR Biomedical Research Centre at University Hospitals Bristol and Weston NHS Foundation Trust and the University of Bristol and NIHR Advanced Surgical Technologies Incubator. The views expressed in this publication are those of the author(s)

For peer review only - http://bmjopen.bmj.com/site/about/guidelines.xhtml

and not necessarily those of the NHS, the National Institute for Health Research or the Department of Health and Social Care. JLD and JMB are NIHR Senior Investigators. NSB is an MRC Clinician Scientist.

Word count: 2978 (excluding title page, abstract, quotes and references)

For peer review only

Abstract:

Objectives: COVID-19 presents a risk of infection and transmission for operating theatre teams. Guidelines to protect patients and staff emerged and changed rapidly based on expert opinion and limited evidence. This paper presents the experiences and innovations developed by international surgical teams during the early stages of the pandemic to attempt to mitigate risk.

Design: In-depth, semi-structured interviews were audio-recorded, transcribed and analysed thematically using methods of constant comparison.

Participants: 43 participants, including surgeons from a range of specialities (primarily general surgery, otolaryngology, neurosurgery, cardiothoracic and ophthalmology), anaesthetists and those in nursing roles.

Setting: The UK, Italy, Spain, USA, China and New Zealand between March and May 2020.

Results: Surgical teams sought to mitigate COVID-19 risks by modifying their current practice with an abundance of strategies and innovations. Communication and teamwork played an integral role in how teams adapted, although participants reflected on the challenges of having to improvise in real time. Uncertainties remained about optimal surgical practice and there were significant tensions where teams were forced to balance what was best for patients whilst contemplating their own safety.

Conclusions: The perceptions of risks during a pandemic such as COVID-19 can be complex and context dependent. Management of these risks in surgery must be driven by evidence-based practice resulting from a pragmatic and novel approach to collation of global evidence.

The context of surgery has changed dramatically, and surgical teams have developed a plethora of innovations. There is an urgent need for high-quality evidence to inform surgical practice that optimises the safety of both patients and healthcare professionals as the COVID-19 pandemic unfolds.

Word count: 266

For peer review only - http://bmjopen.bmj.com/site/about/guidelines.xhtml

Summary box – strengths and limitations

- Undertaking qualitative interviews provided important and rich insights into surgical teams' experiences of current practices in the United Kingdom (UK) and internationally.
- Purposeful sampling ensured that a range of participants were recruited in relation to role, specialty, and geographic location.
- Participants were predominately identified via snowball sampling, making it possible that the findings may not be representative of all healthcare professionals involved in the management and treatment of patients undergoing surgical procedures.
- Interviews were conducted during the first wave of the pandemic, so further research is warranted to continue to explore how teams continue to adapt.

For peer review only

Introduction:

The World Health Organisation declared the COVID-19 outbreak a pandemic on 11 March 2020(1). Many countries have since struggled with escalating case numbers and strained healthcare systems(2). Implementation of infection prevention and control is crucial to deliver healthcare, especially for the personal protection of healthcare workers(3, 4). Operating theatres are particularly vulnerable areas due to high-risk transmission activities such as airway management transmission, aerosol-generating procedures (AGPs) and the involvement of multiple staff(5). The additional strain presented by a high prevalence of disease, limited resources and staff under pressure greatly increase the risks of transmission and the burden on healthcare systems(6). Guidelines were rapidly published (7) (8), including recommendations from The Royal Colleges (such as the Royal College of Surgeons of England, Royal College of Anaesthetists and The Intensive Care Society), Public Health England and the Department of Health, the American College of Surgeons, and the World Health Organisation. These have been produced based on expert opinion, surveys, consensus work and rapid reviews, and continue to evolve as evidence unfolds and can include contradictory recommendations (9-13).

The pandemic inevitably provoked creative solutions to mitigate the risk of infection transmission(14). Innovation could involve: modifications to decisions around whether and how to operate, surgical techniques, tools and technology, surgical team composition and operating conditions (15). Although a handful of studies have suggested practical strategies to mitigate COVID-19 risks (2, 9, 16-19), qualitative inquiry is a valuable tool for capturing dynamic and complex responses to a pandemic (20). These methods allow us to understand the ways people make sense of what is happening around them (21).

The study aimed to explore surgical teams' experiences of current practices during the pandemic. Specific objectives were to i) understand healthcare professionals' perceived risks of COVID-19 in surgery; and ii) to explore how surgical teams mitigated these risks.

Methods:

Semi-structured interviews were conducted with surgical teams in the United Kingdom (UK) and internationally. This qualitative study adopted principles and techniques described by Glaser and Strauss (22) which enabled the inductive identification of codes from the data to generate findings that were grounded in the data (23) and the constant comparison approach, where new findings are systematically compared with existing data so that similarities and differences can be identified and

For peer review only - http://bmjopen.bmj.com/site/about/guidelines.xhtml

emerging theories refined through the ongoing assimilation of data (22, 24). Ethical approval was granted by the University of Bristol Faculty of Health Sciences Research Ethics Committee (reference: 56522), and an amendment enabled data collection to be conducted via University-approved video conferencing software and for interviews to be conducted internationally. Standards for Reporting Qualitative Research (SRQR) guidelines (25) were followed (Supplementary File 1).

Patient and public involvement

Public and patient involvement was not conducted as part of the current study.

Recruitment and sampling

Healthcare professionals involved in the management and treatment of patients undergoing surgical procedures were eligible. A key informant sampling approach was initially adopted(26), whereby individuals from different surgical specialities who were known to the study team were approached. Subsequent participants were identified via snowball sampling(26), whereby interviewees recommended potential participants. Participants were also identified through convenience sampling (27), whereby study details were circulated to members of surgical groups (the National Institute for Health Research Surgical MedTech Co-operative and the Royal College of Surgeons of England). The adverts included instruction to contact the study team if individuals were interested in study participation. As data collection continued, sampling became increasingly purposeful with a view to achieving a sample of maximum variation, to ensure insights were captured from a range of informants operating in different contexts (27). We aimed to capture variation in relation to role (including surgeons, theatre nurses and anaesthetists), specialty, years in profession, gender, and geographic location. Capturing an international perspective was important to identify how best practice evolved, particularly in those areas/nations that were 'ahead of the curve'. A database of participants and their characteristics was maintained and assessed as the study progressed, and interviews with those who were underrepresented were prioritised.

Data collection

Participants provided written consent to take part in the study. Interviews were conducted either via University-approved secure video conferencing software or if not possible, telephone interviews were conducted instead. Interviews were audio-recorded using an encrypted audio-recorder or videoing conference software. Interviews were conducted by experienced qualitative researchers (DE, MJ, LR, CO, KA, SP, JLD). See Supplementary File 2 for further information on the qualitative researchers' backgrounds.

For peer review only - http://bmjopen.bmj.com/site/about/guidelines.xhtml

Semi-structured interviews were directed by a topic guide to ensure that the same core areas were consistently covered amongst the team of interviewers, whilst allowing flexibility to pursue the detail that was salient to each participant (Supplementary File 3). Specific questions were open-ended to encourage the participant to talk about their own experiences of particular topics (“In your own words, can you talk me through how PPE has been used during COVID-19?”), experiences (“Can you describe a case where COVID-19 affected what happened in the operating theatre?”) and views (“In your opinion what are the key risks in surgery due to COVID-19?”). Interviewers were then able to incorporate the interviewee’s own terms and concepts into subsequent questions to follow up on specific issues raised and check the interviewer had understood correctly (28). At the end of the interview, participants were given an opportunity to raise relevant issues that had not already been covered (29). Regular team meetings allowed for the team to review the topic guide in light of findings and consider potential changes (e.g. addition of topics or rephrasing of questions).

Reflexivity is an fundamental part of ensuring the transparency and quality of qualitative research(30). Interviewers’ reflexive notes, which took into account their observations, thoughts and ideas(31), were shared with the analysis team. Regular team meetings also enabled the interviewers to reflect on the novel process of conducting virtual interviews (a form of functional reflexivity, whereby researchers give critical attention to the way processes influenced research(32)).

Data analysis

Interviews were transcribed verbatim, checked for accuracy and de-identified. Transcripts were imported into NVivo (QSR International, USA), where the contents of the transcripts were iteratively coded by three coders (MJ, LR and CO). Codes were identified inductively using methods derived from grounded theory methodology(22, 33), so that findings were grounded in the data to capture and represent healthcare professionals’ interpretations and experiences of surgical practice during a pandemic. Through the constant comparison technique, the codes were categorised into themes which were continually compared with the data(34). In this way, similarities and differences can be identified and emerging themes refined through the ongoing assimilation of data.

A premise of grounded theory is that these comparisons enable identification of ‘negative cases’ (i.e. participants whose perspectives/experiences differed from the main body of evidence)(35, 36). This was felt to be important for enhancing the credibility of the analysis as it helped to ensure the findings represented as full and comprehensive an account of participants’ experiences and perspectives as possible, rather than those views that were dominant or fit with a particular

For peer review only - http://bmjopen.bmj.com/site/about/guidelines.xhtml

impression of the results(22). Data collection continued until no new key themes were identified (data saturation) as determined by the study team.

The coders met regularly to compare their coding and agree on the main broad categories encapsulating all codes. A subset of the transcripts, batched according to coder, were independently double coded by DE to determine broader consistency in coding approaches (37). Descriptive reports were written on key themes (‘Guidance’, ‘Risk’, ‘In-theatre processes’, ‘Organisational’, ‘Service provision’, Personal protective equipment (PPE), ‘Testing’ and ‘Physical and mental impact’). This paper focuses on the findings relating to ‘Risks’, although themes and sub-themes were often intertwined and producing descriptive reports provided an opportunity to demonstrate where there was overlap in key findings. Two coders (CO, MJ) also produced a summary on one theme, to explore any major differences in interpreting codes. As multiple qualitative researchers conducted the interviews, one researcher (DE) read all transcripts and synthesised the findings to ensure consistency in analysis and to enable strategic oversight of the findings(37). Results, with reference to the raw data, were discussed with members of the wider study team (including academic surgeons) and several research participants who were not part of the study team.

Results:

Of the clinicians contacted by the team, nine individuals did not reply to the study invite, said that they were not on the frontline or were unable to find the time for an interview. The final sample of 43 participants included 34 surgeons, five anaesthetists and four individuals in nursing roles (a practice educator, nurse manager, matron and charge nurse). Surgical specialties included general surgery (n=14), otolaryngology (n=11), neurosurgery (n=5), cardiothoracic surgery (n=2) and ophthalmology (n=1). Thirty-one participants were from the UK, and the remaining 12 participants were from Italy (n=3), China (n=1), Spain (n=3), the USA (n=2) and New Zealand (n=3). Participants were mostly male (77%). Interviews were conducted between March-May 2020 and lasted an average of 47 minutes (range=21-116 minutes).

Five sub-themes relating to risk were identified: ‘Facing new and uncertain risks’, ‘Changes to the context of surgery due to COVID-19 risk, ‘Innovations within high risk specialities, ‘Aerosol generating procedures: Complex and uncertain risks’ and ‘Adapting to PPE-related challenges’. Results are supported with detailed quotations to support the interpretation of data (30, 31). Quotes that were deemed to best illustrate the themes were selected, with careful attention to showcasing different perspectives and negative cases, where relevant.

For peer review only - http://bmjopen.bmj.com/site/about/guidelines.xhtml

Facing new and uncertain risks

All informants described how COVID-19 had had an impact on surgery. At the time of interviews, most elective procedures had been cancelled or postponed and only emergency procedures were being performed:

> *HP30: We are doing absolute emergency. … life and limb only... Even to the extent that some appendicectomy operations are being managed conservatively. So, they're just given antibiotics and watch and wait. We know that a lot of people with appendicitis … if they're managed conservatively actually do fine. And a lot of our appendixes now, … are being managed conservatively and watched. I think if you'd suggested that a year ago, people would have said was insane.*

Participants articulated anxiety about contracting the virus. One individual had personally lost a colleague to COVID-19. Many others described how news of clinical professional fatalities had highlighted the sense of personal risk they and their colleagues were being subjected to.

> *HP7: It is just the shock of my life; I had no idea this was coming. I had no idea that walking into the hospital would be dangerous for me... A huge change in my mindset required to deal with this, because it's changed, almost like a war. Like it is normal one day and then within a week, your normal working life is sort of totally changed. Overnight, things changed…it was in the news, we knew it would happen, but then all of a sudden it was here, we were in it.*

Whilst they were accustomed to considering risks for patients, interviewees reflected on the shock of how they suddenly faced new risks to their own health. The risk of transmission to family members added a unique dimension to the risks associated with their roles as healthcare professionals. This was described across a range of surgical specialities and roles.

> *HP4: And the commonest thing about this whole thing, amongst workers, doctors, nurses, everyone: actually, everyone's worried about catching it. Everyone is terrified of giving it to their family […] I don't want to get it. I really, really don't want to get it. But I'm infinitely more scared about giving it to my wife or my children. And if I catch it, they'll get it. Almost certainly. Because I'll be infected for several days before I'm symptomatic […] We're not used to processing the risk to us… Because these are unfamiliar risks in our practice… The risks that we've always worried about in the past were risks to our patients, or risks to our career. Rather than risks to our health. And as I say, more particularly risks to the health of our families.*

Changes to the context of surgery due to COVID-19 risk

COVID-19 had meant that surgical teams had to rapidly assess all aspects of the patient pathway to minimise the potential spread of the virus. For most, the scale of organisational changes was

For peer review only - http://bmjopen.bmj.com/site/about/guidelines.xhtml

immense and included changes to hospitals, pathways, staff rotas and operating theatres. There was an urgent need to expand the capacity for intensive care, with comprehensive changes that often meant re-purposing operating theatres. There were also considerable discussions about changes to accommodate COVID and non-COVID areas, including separate operating theatres for COVID-19 infected patients and those not infected. These changes required new thinking about patient pathways and processes in operating theatres. Considerable effort went into minimising contact points and the number of people in the operating theatre to avoid potential infection transmission and contamination.

> *HP5: It is a massive move. Everyone will look at the pathway for every scenario. We are involving everyone and sometimes you are thinking 'Oh yes we have done the pathway now' and then you realise 'oh we missed that one important point. Can we walk through it again?'.*

The magnitude of these changes meant that teamworking and communication became even more integral, and many participants described the value of ongoing team discussions with a specific focus on COVID-19 related issues. Technology such as video conferencing software or messaging groups had played a huge role to facilitate this.

> *HP3: We were all social distancing at this point and we were having discussions mainly over Microsoft Teams… it's much better now. We started running our cancer MDT on Teams too, that was pretty effective, and we managed to get pretty much the same amount of collaboration or potentially even more because it's quite convenient to be able to dial in from anywhere… I think we'll probably continue to use it in some way all the way into the future hopefully.*

Informants reflected on the challenges of having to adapt as best they could with only limited evidence to guide them (Table 1). One anaesthetist pointed out the potential implications of *ad hoc* innovations in this unusual time:

> *HP4: One of the most dangerous things in medicine is what's called the MacGyver bias. Which is where people believe that their ability to, to fudge a solution out of something will be much better than the usual way of doing it. I think you always have to be aware that, whilst it's important to adapt and to - in this sort of situation particularly you're having to innovate and change the way you work and change the way you do and change the way you approach things. Always be aware that, if you're the first person to come up with a way of doing it, the chances are that a lot of other people will have thought of that before and decided it's a bloody stupid idea overall.*

For peer review only - http://bmjopen.bmj.com/site/about/guidelines.xhtml

Table 1: Learning lessons the hard way

Reflecting on optimal PPE use in patients:<br>*HP40: We're doing things which may be putting ourselves at more risk than non-risk, because they seem to be sensible at face value, but maybe, are not.. We've been asking patients to wear face masks at the time of surgery… as soon as that happened, the lens we were using for the surgery was steaming up every few minutes. So I thought, "Well, if it's steaming up, it must mean that the patient's breath is coming up the mask and out of the drape that we've put on and into the surgical field." So actually, paradoxically, putting a mask on the patient was probably increasing my exposure in the surgical field, rather than decreasing it. But intuitively, everyone thought that if we put a mask on a patient during surgery, we were reducing our risks… So from a protection perspective, we're still learning, I think. There's no doubt that we will be better prepared for the next wave that is being predicted.*
Organisational lessons:<br>*HP4: We turned the top floor into an intensive care unit. And when that started filling up, we realised it was just a really bad idea. Because, although it's a nice idea having patients isolated in separate rooms, if a patient is in a separate room, they need one nurse to look after them in each room. And so, it's very nurse heavy. Whereas, actually, once you start getting a lot of patients, what you need is to be able to cohort the patients. So, 3 or 4 in a bay. And you can have 1 or 2 non-skilled nurses - you know, not ITU-skilled nurses - in there being supervised by 1 ITU nurse. So, you can dilute your skills… The other thing with that plan, the idea was, as our number of patients increased, we could keep them all in the same block and we would just expand intensive care down from the top floor, to the floor below, to the floor below that but unfortunately … there was only an adequate back-up power supply to the top floor. And the next 2 floors below had nowhere near enough back-up power. So, if the mains power went down, we'd kill everyone on the ward instantly. So, we've had to abandon that completely and decant an entire intensive care unit to somewhere else and open a new one up. And build another intensive care unit. Because one of things no-one had thought to check was, actually, was there enough power there.*
Optimising pathways:<br>*HP4: Our plan was always going to be we would collect the patients take them to intensive care, anaesthetise them there and do everything there. But we very swiftly realised that that actually meant a trip with a potentially very unstable patient, through the hospital, not in a controlled situation. We risk contaminating what may be clean bits of the hospital. And so then we actually moved to completely just doing everything on site. So, we essentially take intensive care to the patients. And that saves a lot of time. We think made the process much safer for the patients. And made the process safe for everyone else in the hospital as well. Because people who were infected were only then being moved through the hospital a minimal amount of time, to decrease contamination risk.*

Innovations within high-risk specialities

There was agreement that healthcare professionals who participated in procedures within the head and neck region were at increased risk of infection. For instance, anaesthetists were at high risk due to aerosolisation when intubating and extubating patients due to direct access or direct instrumentation of the airway. There were many strategies adopted in these contexts (Table 2).

Table 2: Modifications to practice for intubating patients

Example	Quotation
**Changes to mode of anaesthesia**	*HP40: One of the innovations that has happened as a direct result of COVID is that surgical procedures that, up until now, we would have said were general anaesthetic procedures have been successfully converted to local procedures… We would never have contemplated doing an enucleation – removing the eye – under a*

For peer review only - http://bmjopen.bmj.com/site/about/guidelines.xhtml

	local anaesthetic before. Now, we have successfully done that.
**Changes to where the patient is intubated**	*HP28: The anaesthetic is being conducted in the theatre whereas normally it would be done in the anaesthetic room in the UK … It just means that they are not moving between lots of different places as well, so they try to minimise the number of points that the patients will stop at.*
**Changes to where patient extubated**	*HP4: Now we would actually wake the patient up in theatre and have them awake in theatre before they went out, where previously, patients would routinely be taken to the recovery area mostly asleep... In the past, where you might have a patient waking up with an airway in, coughing it out, coughing and spluttering a bit. But actually if they do all that whilst we're in the theatre environment, which is already contaminated, and we're all in protective kit.*
**Reducing aerosolisation during intubation**	*HP34: If you put a plastic sheet over the head of the patient that reduces much of the aspiration, so the spit, coming out and then the aerosolisation which causes spit will sit on that plastic more than hit you in the face or the neck or anything like that. Those are good things that came out and you can actually see it, the one I saw, you saw all of the spit on the patient, when you extubate, the spit went all over the patient, if you didn't have that plastic sheet, that would be, because your neck is still exposed and all of that.*
**Pausing to allow aerosols to be dissipated**	*HP17: There's a delay between the patient being put to sleep and the operation being started, just to allow the air to be changed in theatres. The rationale behind that being that when someone's put to sleep and they've got a breathing tube put down, the virus might be kind of put into the air, they might be floating around. Operating rooms have got incredibly high air flow, the air is changed-, it depends on your particular operating theatre but each individual hospital will know how long it takes to cycle the air out of their theatre, and then they can do a bit of maths to see how long it takes for the air in there to be entirely fresh. It's normally about 20, 25 minutes.*

Media coverage showing deaths of ENT surgeons appeared to have impacted on the surgical community's perception of risk for this group, and many described how it made them scrutinise their own practice. Some of the ways that tracheostomies, for example, were adapted to try to mitigate this risk are outlined in Table 3.

> *HP10: Basically the highest concentration of virus in an ill Covid patient is in anything that comes in and out of their mouth and nose, if they've got Covid contains the highest concentration of viral particles with the greatest risk of cross infection to somebody looking after that patient. Very sadly, the first Doctor to die in the UK was an ENT surgeon and they spend their entire lives examining peoples' upper aerodigestive tract and therefore they were at particularly high risk.*

Table 3: Modifications to forming a tracheostomy

Example	Quotation
**Being performed only by a small team of experts**	*HP43: Early on, I was interested in working [on tracheotomies] and a surgeon was interested in it as well. Our personalities work reasonably well together and we started, from the surgical side at least, combining forces and trying to figure out a way to do this safely… We weren't exactly sure which patients were going to make the most sense. We didn't know who exactly would benefit from it, but there was little question there were going to be some people … We go into the room, we wheel in all our stuff. We don't even talk that much anymore. Teamwork is very*

For peer review only - http://bmjopen.bmj.com/site/about/guidelines.xhtml

	crucial… I think it has been important, both from an operational standpoint, but also from a mental support and enthusiasm standpoint. I think, if one of us was doing this alone, without the other, I think it would be a lot more difficult. I wouldn't say it's undoable on your own completely, but it's just crazy to try to do that. You have to ask for help.
**Pausing the ventilator**	*HP41: I think a tracheotomy is more risk… Before we cut open, cut open the trachea, we make the ventilator pause for a second. Then first we pause the ventilator, then we cut, and then we insert the intubation quickly, and we restart the ventilator … If the ventilator is open, then there are a lot of aerosols… Secretion maybe comes out from the lungs, from the bronchia. So if we pause the ventilator, maybe they can decrease the secretions.*
**Location of tracheotomy**	*HP41: All the tracheotomy is at the bedside. So it's including a resident, to help at the head side of the patient, and two surgeons. One is at the bedside, to do the surgery. And one or two nurses help to deliver some things to help the surgery. Now, the unit is the ICU unit. There's one person per room.*

Aerosol generating procedures: Complex and uncertain risks

Participants described how new national and local guidelines had stated that AGPs such as laparoscopic and endoscopic approaches subjected staff to elevated risk. However, many reflected that there had been some uncertainty as to precisely what constituted an AGP within their surgical teams. There were also conflicting views as to whether, in line with guidance, alternative techniques should be adopted.

> *HP13: It's really uncertain. We're bombarded by information from national societies, international societies, there's webinars left right and centre, and I've certainly taken part in a fair few of those to try and understand peoples' perspectives. And we've got that sort of headline idea that surgeons are at risk from aerosolised procedures, but we don't really have any knowledge of what an aerosolising procedure is.*

When contemplating whether to proceed with AGPs, teams weighed up many factors, including the potential risks to the patient, existing evidence (or lack thereof), and what colleagues or those in the wider surgical community were doing. Taken together, informants' accounts suggested that the issue of AGPs created conflicts between their clinical roles where they had to weigh up what was best for their patients whilst contemplating their own safety.

> *HP19: It's not all about saving the physician and the team, it's about everybody in the process and understanding that these are really really, difficult decisions… They're not black and white, they're grey… It's about protecting surgeons, it's not about protecting patients... I'm not sure there's been another example where it's been so clear that effectively national guidance has come out to say you put yourself ahead of the patient care, which is how I interpret the guidance that came out.*

For peer review only - http://bmjopen.bmj.com/site/about/guidelines.xhtml

As the quotes below highlight, informants were conflicted as to how comfortable they felt about putting their own safety ahead of the patient's.

> *HP28: We have to accept risks to us because the patient needs an operation far more than we need to be protected… You look at the newspapers and the clinicians and the nursing staff and the porter staff that have all fallen foul of this, people are going to continue to work, there's an inherent dedication to achieving a safe outcome for a patient if that necessarily places you at risk as well.*

> *HP9: Although you want to do everything for your patient, your own safety and the safety of your team has to come first because if you have a whole team who is not properly protected and everybody gets infected you have taken out a team or an individual of that team for a period of time, so they can't help with the future patients.*

When proceeding with AGPs, participants described adopting the highest level of PPE and implementing strategies and innovations to try to minimise the spread of the virus during aerosolisation (Table 4).

Table 4: Modifications to AGPs

Examples	Quotation
**Wearing full PPE**	*HP28: We would use keyhole surgery and we use various adaptations for the keyhole …we did everything with PPE at the time we saw the patient, full PPE in theatre […] We went against guidance and used keyhole surgery because we felt that there was no evidence to suggest that it's any more risky.*
**Use of smoke-filter evaporators**	*HP21: We use filters to avoid aerosols, every port is connected to a filter so no aerosol comes through the port... We fix the port to the skin to avoid accidental removal, apply a stitch to the port and fix it to the skin, so the chances of the port being accidentally removed is really decreased.*
**Plastic sheets**	*HP12: We cover the head and the drill with this plastic sheet that aerosols are not going into the air, and not going everywhere, to protect the environment and to protect the surgeons.*
**Drapes**	*HP18: We're using some simple things like some plastic drapes over the top of the really aerosol generating stuff that we're doing, making some holes in those drapes so that we can still use our normal instruments.*
**Adopting a combination of approaches**	*HP20: Tracheostomies were done percutaneously … They have done some modification, doing some combination between open and percutaneous tracheostomy…to minimise as much as possible the risk of leaking aerosol in the operative field to the operative team.*

Adapting to PPE-related challenges

At the time of interviews, most participants felt that the PPE provided was sufficient, although some interviewees expressed their concerns about not having access to adequate PPE and described having to outsource their own or 'pushing' their hospitals to provide more or fuller PPE. Moreover, several informants reflected on the news coverage that there had been limited PPE available to

For peer review only - http://bmjopen.bmj.com/site/about/guidelines.xhtml

hospital staff, and expressed concerns that this would happen to them as the pandemic progressed. As guidance emerged stating that healthcare professionals should not put themselves at risk if they did not have sufficient PPE, many were again forced to consider whether to put their own safety before their patients':

> *HP4: Bottom line is, I have a wife and children […] I am not going to put my family at risk by doing something without the right kit. Sorry. That's the way it is. If I haven't got the equipment to do it, I'm not doing […] There's national guidelines on what we should wear for each situation. And if we haven't got it, I'm not doing it.*

Some participants also expressed concern as to whether their PPE was fitted correctly, and teams developed local strategies to facilitate optimal PPE donning/doffing. This included creating visual aids (checklists, signs/posters in theatre), having 'buddy systems' and engaging in team simulations to practice and contemplate different donning/doffing scenarios. Most informants reported that wearing PPE was hot, distracting and uncomfortable, although there was a clear sense that the discomfort was a necessary compromise for sufficient protection.

> *HP3: I am just constantly thinking, have I done this right? Am I putting this on in the right way? Is my mask fitting properly? I just don't know, and then the real feeling of… I don't want to, I really don't want this on my face during this procedure, and am I doing the right thing. And I think one of the things for me is that I don't really feel like we have had very good instructions on how to do these things. So, one of the things that hit me this morning was, nobody really seems to know, nobody seems to be in control, or what's going to happen and these are the processes, this is where you put your stuff. So when you are putting it on and taking it off etc etc, it is a bit chaotic and that makes me really nervous… I hope I got it right, but I don't know.*

Wearing PPE in the operating room also created practical problems that had the potential to impact performance during surgery. The time taken to don PPE was considered problematic in emergency circumstances. One strategy for enhancing efficiency was to pre-pack PPE in 'grab and go' bags to enable staff to proceed more swiftly to theatre. PPE made it significantly harder to communicate between staff in theatre and between staff positioned in 'clean' and 'dirty' areas. Staff were also unable to obtain additional surgical equipment whilst in the operating theatre. Consequently, many participants described strategies to facilitate communication (Table 5).

Table 5: Problems caused by PPE and potential strategies to overcome these issues

Issue caused by PPE	Quotation	Potential solution	Quotation

For peer review only - http://bmjopen.bmj.com/site/about/guidelines.xhtml

**Time taken to don PPE**	*HP19: It's made the operations much longer, by the time you get the kit on.*	**'Grab bags'**	*HP4: We've set up a fantastic production line of grab bags. So, everything we need is in one of a series of grab bags. […] We're going off to intubate someone, so we want that bag and that bag. We're going to put lines in them, so we want that bag.*
**Difficulty determining who is who**	*HP3: You can't take name badges in.*	**Name and roles written on PPE**	*HP11: People do write their names on their gowns or aprons… The problem is you don't know what their role is. They should actually write, 'ITU Nurse'.*
**Difficulty communicating whilst wearing full PPE**	*HP11: I could barely hear anybody. It was really hard.*	**Using equipment to facilitate communication**	*HP34: Vocera is a communications system that you can have clipped on you or if you're in the scrub role you can have it on the side and you can activate it by clicking it. With the N95 mask you don't hear people. If you have Vocera then you can speak and people can hear.*
**PPE fogging up glasses**	*HP19: When I was wearing my spectacles when I was breathing out the air was misting up my spectacles.*	**Masks over glasses**	*HP20: We have been working on the last week on a modification. You have seen some reports in different countries about the use of a snorkel mask.. and it seems to work much better, without fogging.*
**Needing additional equipment**	*HP3: Normally if we need something extra during the operation, someone just walks out of theatre, get its and then comes back in again, but we are not allowed to do because there are clean areas and dirty areas.*	**Equipment to aid communication with 'runners'**	*HP8: Well I brought some walky-talkies which are quite good which help with communicating from inside to outside of the room. So that is one problem. They are quite useful… the runner outside will have one and we have one in a plastic bag in the theatre. I actually bought them on eBay and everyone wants more now so I am buying some more which should arrive any day…. Shouting through the door or holding up signs is difficult. These work quite well actually.*<br>*HP3: One of the anaesthetists had the idea of having some Bluetooth speakers, so one in the clean area and one in the dirty area.*
**Long periods wearing PPE**	*HP34: We realised people can't do nine hours in a theatre with PPE on, wearing the N95 puts incredible pressure on people's faces.*	**Changes in staffing**	*HP35: We have to do a team change, so we changed how we did things there as well. We started looking at team changes and how we would manage those, we'd send people home, knowing that in 4 hours, they'd be coming in to do a team change.*

For some, PPE affected how operations were performed. Surgeons described, for example, how wearing two sets of gloves affected their dexterity and how using a microscope was difficult whilst wearing a visor. These participants described removing their PPE to complete specific tasks. The

For peer review only - http://bmjopen.bmj.com/site/about/guidelines.xhtml

issue of surgeons having to weigh up being fully protected by PPE and their ability to operate once again demonstrates the tension between the safety of patients and healthcare professionals.

> *HP16: The problem surgically with wearing the visors are that the visors sticks out from your face, so I desterilise the instrument on the visor and I desterilise my gloves and the visor so then at the end of the procedure I ended up just having to take the visor off because it was a sterility risk for the procedure I was trying to perform. Trying to use the operating microscope with the visor or with googles on outside of my glasses is just not possible. So at that point, I have areas of my face exposed while I am looking down the operating microscope puttering away at a tumour over creating aerosols whilst not fully covered and I can't be fully covered, else I can't do the surgery if I am.*

Discussion:

Summary of findings and lessons learned

This study captures how surgical teams were suddenly faced with new and unfamiliar risks due to COVID-19. We report how they made sense of and adapted to these risks as the pandemic unfolded. Informants described how there was a plethora of evolving – and sometimes contradictory – local and national recommendations about providing surgical care safely during the pandemic. Our study highlights that guidelines were not a panacea for surgical practice, and at times, there were tensions between clinical roles and personal safety.

We found that communication and teamwork were crucial to how teams adapted. Within this, we identified several practices that appeared helpful for reducing risks and optimising surgical practice during the duration of this pandemic (and potentially other adverse situations). Firstly, healthcare professionals articulated the importance of ensuring all aspects of the preoperative, operative and postoperative pathway was carefully mapped out to identify potential risks and staff roles. This enabled teams to minimise contact points and the number of people in the operating theatre to avoid potential infection transmission and contamination. It is important to note that pathway is likely to need to be reviewed frequently, in light of emerging evidence, experiences and concerns. To ensure practice is dynamic and cohesive, several participants in this study highlighted the value in ensuring all members of the surgical team were communicating regularly to discuss and reflect upon emerging evidence, guidance, experiences and concerns. Participants in our study also conveyed the importance of working together and developing local strategies to ensure PPE is donned and doffed safely and efficiently. This included visual aids (checklists, signs/posters in theatre), having 'buddy systems' and engaging in team simulations.

Implications of findings

For peer review only - http://bmjopen.bmj.com/site/about/guidelines.xhtml

Surgical teams sought to mitigate the risks by modifying their current practice with multiple strategies and innovations to deliver patient care that they felt comfortable with. Nonetheless, uncertainties remained about optimal strategies to minimise risks. Although the epidemiology of COVID-19 has been well reported, there is little robust evidence regarding safe and best surgical practice (38). Our study supports an urgent need for high-quality multicentre research (17), particularly as the pandemic continues to disrupt health services around the world. It has been argued that with the constraints of time and resource, clinical practice must be driven by a pragmatic and novel approach to collate global evidence and to inform evidence-based practice (38).

This study has also highlighted that COVID-19 has had a significant impact on healthcare professionals. This includes experiences of fear and anxiety about personal safety, the challenges of making sense of multiple guidelines that changed frequently, an evolving workload and wearing more PPE. This is supported by findings from interviews with fourteen surgeons from a hospital in Ireland, which reported how COVID-19 had fundamentally impacted clinical roles and had consequences on wellbeing (39). It has been argued that the surgical community need to be provided with support to limit the inevitable psychological burden and risk of burnout (40-44). Shanafelt and colleagues also emphasise that visible leadership is crucial during this turbulent time, and that leaders will need to establish innovative ways to engage with their teams to fully listen to and understand the sources of concern, and work with them to develop approaches that mitigate concerns (45). Interventions must determine how best to support healthcare professionals to process and navigate the physical and psychological repercussions of the pandemic.

Strengths and limitations

The study used qualitative methodology to provide new insights into perceived risks in surgery in the context of COVID-19. Measures were undertaken to ensure rigour of the data in ensuring findings reflected the meaningful impact of COVID-19 on surgical teams. Rigour in qualitative research is determined by a range of criteria(31). Having multiple experienced qualitative methodologists conducting the interviews, analysis and write up enabled analyst triangulation to strengthen credibility of the study (31, 37, 46). To ensure consistency in analysis one individual acted as an 'analytical auditor' and reviewed each transcript to enable strategic overview of the study findings and review the data for discrepancies or overstatements (30, 33, 46). Emerging themes, with reference to the raw data, were discussed with wider members of the study team (including academic surgeons), as well as research participants who were not part of the study team, to ensure

For peer review only - http://bmjopen.bmj.com/site/about/guidelines.xhtml

For peer review only

a good fit between researchers' interpretations and representation of the participants' experiences (30, 31, 47).

Our study had some methodological limitations. Initially, individuals who were known to the study team were approached to consider participation and subsequent participants were identified via snowball sampling. It is therefore possible that the findings may not be representative of all healthcare professionals. The researchers in this study were known to have a particular interest in surgical innovation and responses may have also been influenced by participants' perceptions of what might constitute socially desirable answers, particularly given the emotive nature of some of the topics discussed in the interviews (e.g. resource constraint, balancing risks to patients versus risks to themselves). However, contrasting perspectives did emerge from our sample, and we sought to search for and report negative cases that did not align with recurring ideas. The findings have been reported with negative cases in mind, to present as comprehensive an account of key themes as possible (22).

It is important to note that interviews were conducted between March and May 2020, which has limitations for the transferability of results. Whilst we were able to capture initial responses as the pandemic unfolded in its first wave, future research is warranted to explore how teams continue to adapt during the later stages and aftermath of the pandemic. There are also limitations in the diversity of countries and professions represented in this study, as the majority of participants were from the UK (76%) and surgeons (83%). Findings may have been further developed with greater representation of non-UK settings, and other professions (e.g. anaesthetists, nurses, operating department practitioners). Moreover, participants were from developed countries. Risk mitigation in surgery may look very different in low- and middle-income countries with fewer resources, different healthcare infrastructures and more recent experience of epidemics. Capturing a wider range of international perspectives would inform the wider discourse on optimal practices for surgical procedures during the pandemic.

Conclusion

In summary, our study provides insights into how surgical teams had to rapidly modify their practice with a wide range of innovations to mitigate the risk of COVID-19. Communication and teamwork played an integral role in how teams adapted. Whilst an abundance of guidance was available, perceptions of risks were complex and context dependent. Taken together, this study highlights that the context of surgery has changed dramatically and that there are many uncertainties about best

For peer review only - http://bmjopen.bmj.com/site/about/guidelines.xhtml

practice. As the COVID-19 pandemic continues to unfold, it is imperative that future research continues to explore how teams adapt and that surgical practice is based upon high-quality evidence that optimises the safety of both patients and healthcare professionals.

Author contributions:

DE was the project lead and developed the study protocol, with all authors reviewing and providing comments on subsequent versions. SC conducted literature searches for the study. DE, CO, LR, MJ, KNLA, SP and JLD conducted interviews. DE, CO, LR, MJ and JLD analysed the data. JMB, MB, NSB, PH, DJ, DE, MJ, LR and JLD identified potential study participants. DE drafted the initial version of the manuscript, with critical revisions from JLD, JMB and LR. JMB, MB, NSB, DJ and PH conceptualised the study design. All authors (DE, CO, MJ, NSB, KNLA, SP, SC, AS, PH, DJ, MB, JMB, JLD, LR) commented on the manuscript and gave approval for the manuscript to be submitted. The NIHR Bristol Biomedical Research Centre Surgical Innovation theme is directed by JMB (theme lead).

Disclosure: The authors declare no conflict of interest.

Acknowledgements: We would like to thank Rosetrees Trust for funding this study, and each participant for taking the time to share their experiences with us. We are also grateful to Murat Akkulak, Steven Beech, Becky Carthy, Jenny Russe, Jane Collingwood, Chris Pawsey and Tom Steart-Feilding for providing help and support with the study.

Ethical approval statement: Ethical approval was granted by the University of Bristol Faculty of Health Sciences Research Ethics Committee (reference: 56522).

Data sharing agreement: No additional data available.

For peer review only

References

1. Organization WH. Coronavirus disease 2019 (COVID-19): situation report, 72. 2020.
2. Tay JK, Khoo ML-C, Loh WSJJOH, Surgery N. Surgical considerations for tracheostomy during the COVID-19 pandemic: lessons learned from the severe acute respiratory syndrome outbreak. 2020.
3. Wang J, Zhou M, Liu FJTJoHI. Reasons for healthcare workers becoming infected with novel coronavirus disease 2019 (COVID-19) in China. 2020.
4. Chang D, Xu H, Rebaza A, Sharma L, Cruz CSDJTLRM. Protecting health-care workers from subclinical coronavirus infection. 2020;8(3):e13.
5. Givi B, Schiff BA, Chinn SB, Clayburgh D, Iyer NG, Jalisi S, et al. Safety Recommendations for Evaluation and Surgery of the Head and Neck During the COVID-19 Pandemic. 2020.
6. Brindle M, Gawande AJAoS. Managing COVID-19 in surgical systems. 2020.
7. Collaborative C. Global guidance for surgical care during the COVID-19 pandemic.n/a(n/a).
8. Mayol J, Fernández Pérez C. Elective surgery after the pandemic: waves beyond the horizon. The British journal of surgery. 2020:10.1002/bjs.11688.
9. Wong J, Goh QY, Tan Z, Lie SA, Tay YC, Ng SY, et al. Preparing for a COVID-19 pandemic: a review of operating room outbreak response measures in a large tertiary hospital in Singapore. 2020:1-14.
10. Heldwein FL, Loeb S, Wroclawski ML, Sridhar AN, Carneiro A, Lima FS, et al. A systematic review on guidelines and recommendations for urology standard of care during the COVID-19 pandemic. 2020.
11. Uwins C, Bhandoria GP, Shylasree T, Butler-Manuel S, Ellis P, Chatterjee J, et al. COVID-19 and gynecological cancer: a review of the published guidelines. 2020;30(9):1424-33.
12. Moletta L, Pierobon ES, Capovilla G, Costantini M, Salvador R, Merigliano S, et al. International guidelines and recommendations for surgery during Covid-19 pandemic: a systematic review. 2020.
13. Byrne H, Chawla A, Gurung G, Hughes G, Rao MJTS. Variations in colorectal cancer surgery practice across the United Kingdom during the COVID-19 pandemic–'Every land has its own law'. 2020.
14. Thornton JJb. Clinicians are leading service reconfiguration to cope with covid-19. 2020;369.
15. Hutchison K, Rogers W, Eyers A, Lotz M. Getting Clearer about surgical innovation: a new definition and a new tool to support responsible practice. Ann Surg. 2015;262(6):949-54.
16. Ti LK, Ang LS, Foong TW, Ng BSWJCJoAJcda. What we do when a COVID-19 patient needs an operation: operating room preparation and guidance. 2020:1-3.
17. Collaborative CJTBjos. Global guidance for surgical care during the COVID-19 pandemic. 2020.
18. Søreide K, Hallet J, Matthews JB, Schnitzbauer AA, Line PD, Lai PBS, et al. Immediate and long-term impact of the COVID-19 pandemic on delivery of surgical services. The British journal of surgery. 2020:10.1002/bjs.11670.
19. Bracale U, Podda M, Castiglioni S, Peltrini R, Sartori A, Arezzo A, et al. Changes in surgicaL behaviOrs dUring the CoviD-19 pandemic. The SICE CLOUD19 Study. Updates Surg. 2021;73(2):731-44.
20. Teti M, Schatz E, Liebenberg L. Methods in the Time of COVID-19: The Vital Role of Qualitative Inquiries. SAGE Publications Sage CA: Los Angeles, CA; 2020.
21. Leach M, Parker M, MacGregor H, Wilkinson AJIoDS. COVID-19—A social phenomenon requiring diverse expertise. 2020.
22. Glaser BG, Strauss AL. The Discovery of Grounded Theory. Aldine Transaction: USA; 1967.
23. Krippendorff KJHcr. Reliability in content analysis: Some common misconceptions and recommendations. 2004;30(3):411-33.
24. Miles MB, Huberman AM, Huberman MA, Huberman M. Qualitative data analysis: An expanded sourcebook: sage; 1994.
25. O'Brien BC, Harris IB, Beckman TJ, Reed DA, Cook DAJAM. Standards for reporting qualitative research: a synthesis of recommendations. 2014;89(9):1245-51.
26. Dempster M. A Research Guide for Health and Clinical Psychology: Palgrave Macmillan; 2011.
27. Patton MQJQsw. Two decades of developments in qualitative inquiry: A personal, experiential perspective. 2002;1(3):261-83.
28. Rubin HJ, Rubin IS. Qualitative interviewing: The art of hearing data: sage; 2011.
29. Brinkmann S, Kvale S. Interviews: Learning the craft of qualitative research interviewing: Sage Thousand Oaks, CA; 2015.
30. Lincoln YS, Guba EGJNi. Establishing trustworthiness. 1985;289(331):289-327.
31. Korstjens I, Moser AJEJoGP. Series: Practical guidance to qualitative research. Part 4: Trustworthiness and publishing. 2018;24(1):120-4.
32. Wilkinson SJJoHP. Focus groups in health research: Exploring the meanings of health and illness. 1998;3(3):329-48.
33. Seale C, Silverman DJTEJoPH. Ensuring rigour in qualitative research. 1997;7(4):379-84.
34. Glaser BG, Holton JJHSRHSS. Remodeling grounded theory. 2007:47-68.
35. Henwood KL, Pidgeon NFJBjop. Qualitative research and psychological theorizing. 1992;83(1):97-111.
36. Shirani F, Henwood KJIJoSRM. Continuity and change in a qualitative longitudinal study of fatherhood: relevance without responsibility. 2011;14(1):17-29.
37. Elliott R, Fischer CT, Rennie DLJBjocp. Evolving guidelines for publication of qualitative research studies in psychology and related fields. 1999;38(3):215-29.

For peer review only - http://bmjopen.bmj.com/site/about/guidelines.xhtml

38. Welsh Surgical Research Initiative C. Recommended operating room practice during the COVID-19 pandemic: systematic review. BJS Open. 2020:10.1002/bjs5.50304.
39. Whelehan DF, Connelly TM, Ridgway PFJTS. COVID-19 and surgery: A thematic analysis of unintended consequences on performance, practice and surgical training. 2020.
40. Spinelli A, Pellino G. COVID-19 pandemic: perspectives on an unfolding crisis. The British journal of surgery. 2020;107(7):785-7.
41. de Pablo GS, Serrano JV, Catalan A, Arango C, Moreno C, Ferre F, et al. Impact of coronavirus syndromes on physical and mental health of health care workers: Systematic review and meta-analysis. 2020.
42. Zerbini G, Ebigbo A, Reicherts P, Kunz M, Messman HJGGMS. Psychosocial burden of healthcare professionals in times of COVID-19–a survey conducted at the University Hospital Augsburg. 2020;18.
43. Dzau VJ, Kirch D, Nasca TJNEJoM. Preventing a parallel pandemic—a national strategy to protect clinicians' well-being. 2020.
44. Cahan EM, Levine LB, Chin WWJNEJoM. The Human Touch—Addressing Health Care's Workforce Problem amid the Pandemic. 2020;383(18):e102.
45. Shanafelt T, Ripp J, Trockel MJJ. Understanding and addressing sources of anxiety among health care professionals during the COVID-19 pandemic. 2020;323(21):2133-4.
46. Smith JAJRmip. Semi-structured interviewing and qualitative analysis. 1995.
47. Guba EG, Lincoln YSJHoqr. Competing paradigms in qualitative research. 1994;2(163-194):105.

For peer review only

Reporting checklist for qualitative study.

Based on the SRQR guidelines.

Instructions to authors

Complete this checklist by entering the page numbers from your manuscript where readers will find each of the items listed below.

Your article may not currently address all the items on the checklist. Please modify your text to include the missing information. If you are certain that an item does not apply, please write "n/a" and provide a short explanation.

Upload your completed checklist as an extra file when you submit to a journal.

In your methods section, say that you used the SRQRreporting guidelines, and cite them as:

O'Brien BC, Harris IB, Beckman TJ, Reed DA, Cook DA. Standards for reporting qualitative research: a synthesis of recommendations. Acad Med. 2014;89(9):1245-1251.

		Reporting Item	Page Number
**Title**			
	#1	Concise description of the nature and topic of the study identifying the study as qualitative or indicating the approach (e.g. ethnography, grounded theory) or data collection methods (e.g. interview, focus group) is recommended	1
**Abstract**			
	#2	Summary of the key elements of the study using the abstract format of the intended publication; typically includes background, purpose, methods, results and conclusions	3
**Introduction**			
Problem formulation	#3	Description and significance of the problem / phenomenon studied: review of relevant theory and empirical work; problem statement	5

For peer review only

Purpose or research question	#4	Purpose of the study and specific objectives or questions	5
**Methods**			
Qualitative approach and research paradigm	#5	Qualitative approach (e.g. ethnography, grounded theory, case study, phenomenolgy, narrative research) and guiding theory if appropriate; identifying the research paradigm (e.g. postpositivist, constructivist / interpretivist) is also recommended; rationale. The rationale should briefly discuss the justification for choosing that theory, approach, method or technique rather than other options available; the assumptions and limitations implicit in those choices and how those choices influence study conclusions and transferability. As appropriate the rationale for several items might be discussed together.	5
Researcher characteristics and reflexivity	#6	Researchers' characteristics that may influence the research, including personal attributes, qualifications / experience, relationship with participants, assumptions and / or presuppositions; potential or actual interaction between researchers' characteristics and the research questions, approach, methods, results and / or transferability	6, 18, 19, Supplementary File 2
Context	#7	Setting / site and salient contextual factors; rationale	6
Sampling strategy	#8	How and why research participants, documents, or events were selected; criteria for deciding when no further sampling was necessary (e.g. sampling saturation); rationale	6
Ethical issues pertaining to human subjects	#9	Documentation of approval by an appropriate ethics review board and participant consent, or	6

For peer review only

		explanation for lack thereof; other confidentiality and data security issues	
Data collection methods	#10	Types of data collected; details of data collection procedures including (as appropriate) start and stop dates of data collection and analysis, iterative process, triangulation of sources / methods, and modification of procedures in response to evolving study findings; rationale	6-7
Data collection instruments and technologies	#11	Description of instruments (e.g. interview guides, questionnaires) and devices (e.g. audio recorders) used for data collection; if / how the instruments(s) changed over the course of the study	6-7
Units of study	#12	Number and relevant characteristics of participants, documents, or events included in the study; level of participation (could be reported in results)	8
Data processing	#13	Methods for processing data prior to and during analysis, including transcription, data entry, data management and security, verification of data integrity, data coding, and anonymisation / deidentification of excerpts	8-9
Data analysis	#14	Process by which inferences, themes, etc. were identified and developed, including the researchers involved in data analysis; usually references a specific paradigm or approach; rationale	8-9
Techniques to enhance trustworthiness	#15	Techniques to enhance trustworthiness and credibility of data analysis (e.g. member checking, audit trail, triangulation); rationale	6, 7, 8, 18, 19
**Results/findings**			
Syntheses and interpretation	#16	Main findings (e.g. interpretations, inferences, and themes); might include development of a	7-17

For peer review only

		theory or model, or integration with prior research or theory	
Links to empirical data	#17	Evidence (e.g. quotes, field notes, text excerpts, photographs) to substantiate analytic findings	7-17
**Discussion**			
Intergration with prior work, implications, transferability and contribution(s) to the field	#18	Short summary of main findings; explanation of how findings and conclusions connect to, support, elaborate on, or challenge conclusions of earlier scholarship; discussion of scope of application / generalizability; identification of unique contributions(s) to scholarship in a discipline or field	17-19
Limitations	#19	Trustworthiness and limitations of findings	18-19
**Other**			
Conflicts of interest	#20	Potential sources of influence of perceived influence on study conduct and conclusions; how these were managed	20
Funding	#21	Sources of funding and other support; role of funders in data collection, interpretation and reporting	1-2

The SRQR checklist is distributed with permission of Wolters Kluwer © 2014 by the Association of American Medical Colleges. This checklist was completed on 12. February 2020 using https://www.goodreports.org/, a tool made by the EQUATOR Network in collaboration with Penelope.ai

For peer review only

Researcher profiles

All individuals who conducted the interviews (DE, CO, LR, MJ, JLD, SP, KNLA) have extensive experience of the application of qualitative research methods to improve the design and conduct of health services research, and each have PhDs in health-related fields using qualitative methods. All researchers are based at the University of Bristol, and are members of the QuinteT research group (which uses qualitative research methods to optimise recruitment and informed consent to randomised controlled trials, many of which are surgical) and of the Centre for Surgical Research (which aims to improve the surgical evidence base, and subsequently patient care, through high quality multidisciplinary research). JLD is a Professor of Social Medicine. DE is a Research Fellow in Qualitative Methodology Research. LR is a Senior Lecturer in Qualitative Health Sciences. MJ is a Senior Lecturer in Qualitative Health Science and Senior Research Fellow. CO is a Senior Research Associate in Health Services Research. KNLA is a Senior Lecturer in Health Services Research. SP is a Research Fellow in Qualitative Methodology Research. All researchers involved in the analysis (DE, CO, LR, MJ, JLD) have experience of conducting grounded theory methodology in multiple projects and have published their findings in peer reviewed journals (see below for examples).

Selected publications from the research team, using grounded theory approaches:

Donovan, J. (1995), The process of analysis during a grounded theory study of men during their partners' pregnancies. Journal of Advanced Nursing, 21: 708-715. https://doi.org/10.1046/j.1365-2648.1995.21040708.x

Barclay L, **Donovan J**, Genovese A. Men's experiences during their partner's first pregnancy: a grounded theory analysis. Australian Journal of Advanced Nursing. 1996 Autumn;13(3):12-24. PMID: 8717683.

Featherston K, **Donovan JL**. Random allocation or allocation at random? Patients' perspectives of participation in a randomised controlled trial. BMJ. 1998;317(7167):1177-80.

Donovan JL, Paramasivan S, de Salis I, Toerien M. Clear obstacles and hidden challenges: understanding recruiter perspectives in six pragmatic randomised controlled trials. Trials. 2014;15(1):5.

Donovan JL, Salis I, Toerien M, Paramasivan S, Hamdy FC, Blazeby JM. The intellectual challenges and emotional consequences of equipoise contributed to the fragility of recruitment in six randomised controlled trials. J Clin Epidemiol. 2014;67.

For peer review only

Elliott D, Hamdy FC, Leslie TA, Rosario D, Dudderidge T, Hindley R, **Donovan JL**. Overcoming difficulties with equipoise to enable recruitment to a randomised controlled trial of partial ablation vs radical prostatectomy for unilateral localised prostate cancer. BJU Int. 2018;122(6):970-7.

Husbands S, **Elliott D**, Davis T, Blazeby JM, Harrison EF, Montgomery AA, Sprange K, Dule L, Karantana A, Hollingworth W, Mills N. (2020). Pilot and Feasibility Studies, 6, 173: https://doi.org/10.1186/s40814-020-00710-1.

Jepson M, **Elliott D**, Conefrey C, Wade W, **Rooshenas L**, Wilson C, Beard D, Blazeby JM, Birtle A, Halliday A, Stein R, **Donovan JL**. (2018). Patients find it difficult to comprehend gambling-related metaphors and computer-agency descriptions of randomisation during recruitment to randomised controlled trials. Journal Clinical Epidemiology. DOI:10.1016/j.jclinepi.2018.02.018

Townsend D (**Elliott**), Reeves B, Taylor J, Chakravarthy U, O'Reilly D, Hogg R, et al. Health professionals' and service users' perspectives of shared care for monitoring wet age-related macular degeneration: a qualitative study alongside the ECHoES trial. BMJ Open. 2015;5(4).

Rooshenas L, Elliott D, Wade J, **Jepson M**, Paramasivan S, Strong S, et al. Conveying Equipoise during Recruitment for Clinical Trials: Qualitative Synthesis of Clinicians' Practices across Six Randomised Controlled Trials. PLoS Med. 2016;13(10):e1002147.

Rooshenas L, et al. Bluebelle study (phase A): a mixed-methods feasibility study to inform an RCT of surgical wound dressing strategies. BMJ open. 2016;6(9):e012635.

Zahra J, Paramasivan S, Blazeby J, Cousins S, Avery K, Blencowe N, Mathews J, **Elliott D**. Discussing surgical innovation with patients: A qualitative study of surgeons' and governance representatives' views. BMJ Open. 2020;10:e035251. doi:10.1136/bmjopen-2019-035251

For peer review only

Understanding risks, mitigation and innovations for Surgery in a COVID19 world: Topic guide

Introduction

- Thank you for taking the time to talk. Know there is so much going on at the moment and will try not to take too long.
- You can stop the interview at anytime.
- Purpose of interview:
    - We know that COVID19 presents new risks in theatre and to operating.
    - Across the world all sorts of ideas are emerging to mitigate the risk and experience is accumulating.
    - The purpose of our interview study is to listen to your **views and to understand your experiences and any solutions you have found to these challenges**.
    - We find that personal views of complex situations may yield information not accessed by standard methods. We're hoping to identify useful ideas and we intend to share good practice from this study rapidly in real time (anonymised data) to help others.
- Am quali researcher at UoB (not HP – apologies if some questions seem silly).
- Everything confidential, quotes anonymous.

Check recording

- Will send/have sent written consent form but can I just confirm you're happy to take part in the interview and for me to record it?

<u>**Impact of COVID19**</u>

How are you? How things have been in hospital since the pandemic?

- Can you talk to me about how Covid19 has affected surgery? *(Consider both elective and emergency procedures).*
- Which procedures are considered 'essential' during this time? *How judged? Who/how decisions made?*
- In your opinion what are the key risks in surgery due to Covid19? How identified these risks? *(training, guidance, experience)*
- Which procedures within your specialty are at a higher risk during this time? Why?

Can you describe a case where COVID19 affected what happens in the operating theatre?

- In Covid confirmed patients, those suspected, or all?
- Which staff at risk?

<u>**PPE**</u>

I'm still getting to grips with what constitutes as PPE. In your own words, can you talk me through how PPE has been used during Covid19? (ie – masks, eyewear, full hazmat suits)

- Thoughts on these in terms of:
    - *Practicalities?*
    - *Comfort?*
    - *Communication?*
    - *Whether training provided/needed?*
    - *Availability?*
    - *Does the equipment provide sufficient mitigation to risks of spread? If not, why not?*
    - *What would you class as minimal/optimal PPE?*
    - *Are there different examples for different procedures?*
    - *Necessary for confirmed Covid19 patients, suspected, or all patients, at this time?*

<u>**Role of team**</u>

How has COVID19 affected the theatre team?

- Are staff self-isolating if COVID19 suspected? Are staff tested?
- The RCS Guidance refers to 'minimal theatre staff'. Has Covid19 affected who is present? *How do you define 'minimal? Who do you think SHOULD be there (essential/ desirable)*
- Has COVID affected theatre time? *Pre/post-op prep?*

<u>**Considering other factors**</u>

What else is important? Is there anything else do we need to know or info we should we share with others? Consider:

- Surgical approaches that may be safer/riskier? *(e.g. laparoscopic, endoscopic, open – and implications of changing techniques)*
- Pre-surgical prep?
- Handwashing?

For peer review only

- Location of surgery *(e.g. anteroom)*
- Affected how anaesthesia used?

Given that so much has changed because of Covid19, how will surgery be in the future?

- How to re-start surgery that has stopped?

Ending the interview

- Summarise key info – what are key lessons learned regarding safety in surgery? Anything else important?
- Thank for time
- Request additional people to interview

For peer review only
